# Dietary factors in relation to hypertension: a mendelian randomization study

**DOI:** 10.1186/s41043-024-00575-7

**Published:** 2024-06-21

**Authors:** Jiasheng Cai, Xiaochen Sun, Mingxuan Li, Rong Luo, Wei Wang, Zilong Wang, Mohammed Ahmed Akkaif, Haibo Liu

**Affiliations:** Departments of Cardiology, Qingpu Branch of Zhongshan Hospital affiliated to Fudan University, 1158 Park East Road, Shanghai, 201700 China

**Keywords:** Dietary factors, Hypertension, Mendelian randomization study, Single nucle-otide polymorphisms, Cardiovascular disease

## Abstract

**Background:**

Observational studies have elucidated the associations between dietary factors and hypertension. Nevertheless, the exploration of these relationships using Mendelian randomization remains scarce currently.

**Methods:**

The Mendelian randomization approach investigated the potential causal relationships between 16 dietary factors and hypertension. To achieve this, we identified genetic variants associated with these dietary factors by utilizing data from European-descent genome-wide association studies with a stringent significance threshold (*P* < 5 × 10 − 8). Subsequently, we obtained genetic associations with hypertension from the extensive FinnGen Study, encompassing 92,462 cases and 265,626 controls. Our primary analytical method was the inverse variance weighted method, and we also conducted assessments for heterogeneity and pleiotropy to ensure the robustness and reliability of our findings.

**Results:**

The study revealed significant associations with hypertension risk for various dietary factors. Specifically, higher weekly alcohol consumption (OR: 1.53, 95% CI: 1.19–1.96) and more frequent alcohol intake (OR: 1.20, 95% CI: 1.08–1.33) were positively correlated with an increased risk of hypertension. Likewise, increased poultry intake (OR: 3.25, 95% CI: 1.83–5.78) and beef intake (OR: 1.80, 95% CI: 1.09–2.97) were also linked to a higher risk of hypertension. Conversely, there were protective factors associated with a decreased risk of hypertension. These included consuming salad and raw vegetables, dried fruits, cheese, and cereals. It is important to note that no evidence of pleiotropy was detected, underscoring the robustness of these findings.

**Conclusions:**

This study uncovered causal relationships between various dietary factors and hypertension risk. Specifically, alcohol consumption in terms of drinks per week and intake frequency, as well as poultry and beef intake, were causally associated with an elevated risk of hypertension. In contrast, consuming salad/raw vegetables, dried fruits, cheese, and cereals demonstrated an inverse causal association with hypertension, suggesting a potential protective effect.

**Supplementary Information:**

The online version contains supplementary material available at 10.1186/s41043-024-00575-7.

## Introduction

The prevalence of hypertension has been on the rise globally in recent years, with strong links to cardiovascular disease (CVD) [1]. The World Health Organization has found that one in five individuals suffers from hypertension worldwide, which means more than 1.3 billion patients with high blood pressure. One-third of patients with hypertension have been attributed to unhealthy diets [2–6], and the regulation of dietary factors may ameliorate pathophysiological mechanisms of high blood pressure and prevent the onset and progression of hypertension. Although observational studies have elucidated the associations between dietary factors and hypertension, it is unclear whether these dietary factors play a direct causal role in the development of hypertension or if they are simply a consequence of shared risk factors. A deeper understanding of which dietary factors have a causal influence can contribute to the identification of potential targets for preventing hypertension and cardiovascular disease.

Utilizing Mendelian randomization (MR) methodology, grounded in genetic instrumental variable analysis, employs single nucleotide polymorphisms (SNPs) as instrumental variables (IVs) to discern and confirm causative links between risk elements and disease manifestations. This technique diminishes susceptibility to biases like reverse causation and confounding variables [7]. Given the proliferation of comprehensive genome-wide association studies (GWAS), MR techniques have gained prominence in probing connections between diverse CVD risk elements—such as smoking, body mass index, alcohol intake, physical activity, and lipid profiles—and the susceptibility to hypertension [8–12].

Although GWAS data focused on dietary factors, more MR framework research is still needed to elucidate associations between these nutritional factors and hypertension. In the current investigation, we employed MR methodology to rigorously scrutinize the causative links between 16 distinct dietary factors and the propensity for hypertension, leveraging the most contemporary and expansive GWAS datasets accessible for this endeavor.

## Materials and methods

### Two-sample MR design

A two-sample MR design was used in the study, which relies on two independent datasets, and the MR analysis was conducted while adhering to several fundamental assumptions. First, the IVs used in the analysis must be strongly and robustly associated with the exposure factors under investigation. Second, these IVs should not influence the outcome through pathways other than the specific exposure of interest. Thirdly, the IVs must remain uncorrelated with potential confounding variables that might influence the relationship between the risk factor and the observed outcome.

For this investigation, we amassed and meticulously examined GWAS datasets from the UK Biobank and FinnGen Biobank. It is crucial to emphasize that, given our utilization of publicly accessible, anonymized, and de-identified data, the present study fell outside the purview of mandatory ethical review or approval by any Ethical Review Authority. Notably, the Department of Cardiology at Fudan University Zhongshan Hospital, Qingpu Branch, supported this research initiative.

### Data sources and selection of genetic instruments

We procured genetic instruments focusing on diet-related exposures, encompassing a broad spectrum of dietary behaviors. These included beverage consumption metrics (such as frequency of alcohol intake, weekly alcoholic beverage consumption, coffee consumption, and tea consumption), meat consumption patterns (encompassing processed meat, poultry, beef, pork, oily fish, and non-oily fish intake), as well as vegetable, fruit, and staple food consumption (which involved raw salad/vegetable, cooked vegetable, dried fruit, cereal, bread, and cheese intake). These genetic instruments were sourced from GWAS, predominantly centered on individuals of European descent [13]. Relevant summary-level data were extracted from the UK Biobank via the IEU open GWAS project [13, 14].

We rigorously applied specific selection criteria in our quest for apt SNPs to encapsulate these dietary factors. Specifically, these SNPs were mandated to manifest a pronounced genome-wide association with their corresponding dietary factors (*P* < 5 × 10^-8), maintain a clumping window extending beyond 10,000 kb, and exhibit minimal linkage disequilibrium (r^2 < 0.001). Moreover, the F statistic associated with these chosen SNPs surpassed the threshold of 10, affirming a sturdy correlation between these instrumental variables and the dietary exposures (refer to Supplementary Table S1).

**Fig. 1:**
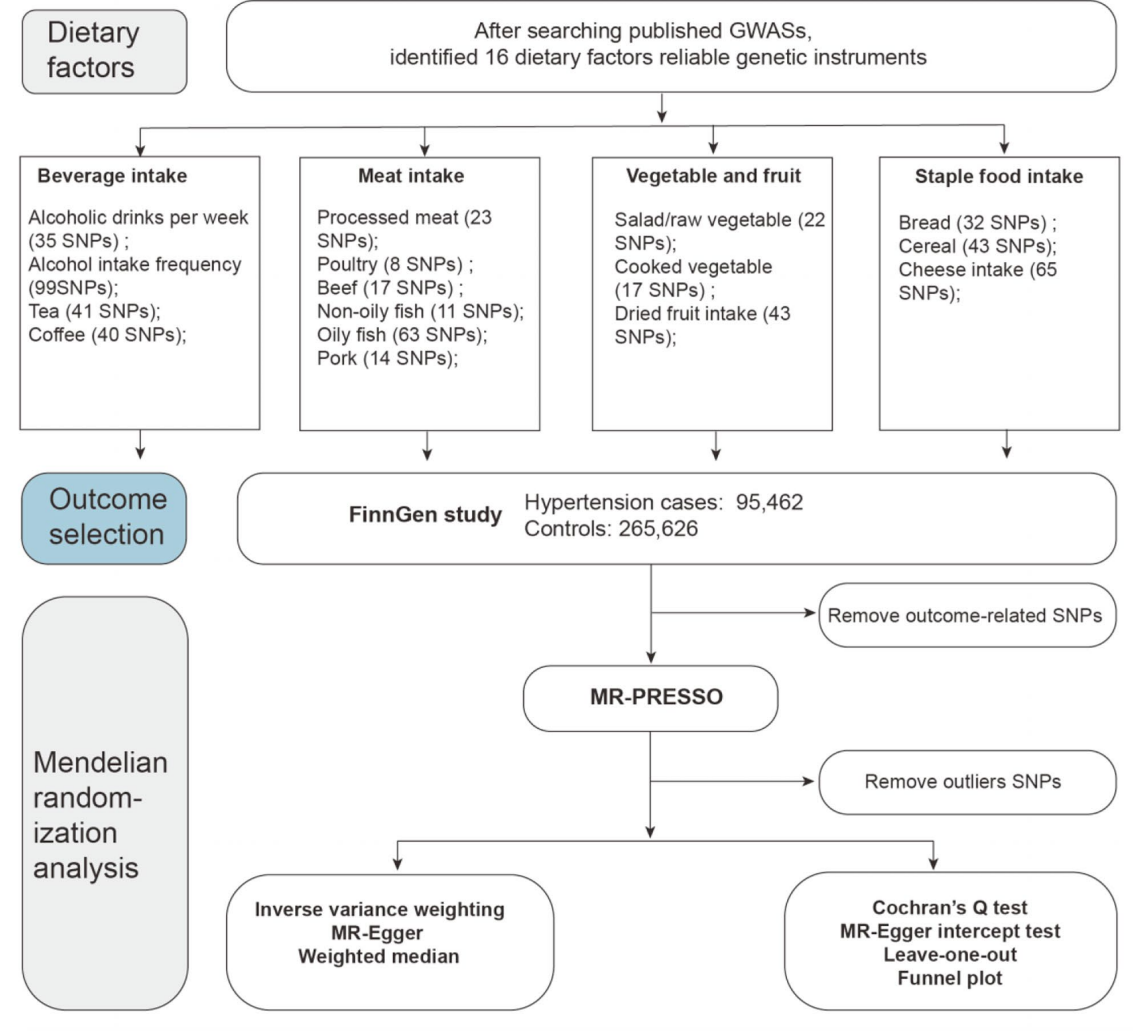
The flowchart of the study design; MR: Mendelian randomization; SNP: single nucleotide polymorphisms; IVW: inverse-variance weighted; MR-PRESSO: MR Pleiotropy RESidual Sum and Outlier

Before executing each MR analysis, we deployed the MR-Pleiotropy RESidual Sum and Outlier (MR-PRESSO) methodology to mitigate potential outliers and address concerns related to horizontal pleiotropy [15]. A schematic representation detailing the analytical workflow is delineated in Fig. 1.

For the hypertension related GWAS data used in our study, we sourced this information from the FinnGen Study [16]. The FinnGen Study is a comprehensive national GWAS meta-analysis involving multiple cohorts and biobanks in Finland. Importantly, the FinnGen Study had minimal overlap with the GWAS data used for exposure assessment. To define and identify hypertension cases in the FinnGen Study, we utilized the International Classification of Diseases (ICD) diagnosis codes. Detailed information regarding the specific ICD codes used can be found in Supplementary Table S2 and on the following website: https://www.finngen.fi/fi.

### Statistical analysis

Several MR methods assessed variant heterogeneity and pleiotropy effects, ensuring robust and reliable results. To achieve this, we first identified and removed outliers using the MR-PRESSO method and excluded hypertension-related SNPs. Subsequently, we applied the following MR methods: Random-Effect Inverse Variance Weighted (IVW): The IVW method was used to calculate the causal effect. This method combines the SNP-exposure and SNP-outcome associations and estimates the overall causal effect. The heterogeneity of the IVW model was assessed using Cochran’s Q test. MR Egger: MR Egger is another approach that improves IVW estimates by allowing for pleiotropic effects across all genetic variants. It assumes that pleiotropic effects are not related to the variant-exposure association [17]. Weighted Median (WM) Method: The WM method is a robust approach that can handle up to 50% of invalid instruments. It provides more robust and consistent estimates, although they may have wider confidence intervals (CIs).

In the IVW analysis context, the intercept is rigorously constrained to zero. Here, the gradient of the weighted regression, elucidating the associations between SNP outcomes and SNP exposure, offers insights into the causal estimate. We deployed Cochran’s Q test with the MR-Egger intercept test to scrutinize heterogeneity.

We leveraged leave-one-out analyses and funnel plots to ascertain potential horizontal and directional pleiotropy further. Moreover, we incorporated forest plots—akin to those utilized in meta-analyses—to visually encapsulate our findings for a more intuitive representation and comprehension of the outcomes. These analytical endeavors were facilitated through the R software (version 4.2.0) coupled with the TwoSampleMR package. This approach was meticulously adopted to ensure a thorough and rigorous examination of the causal interplay between dietary factors and the risk of hypertension.

## Results

### SNP selection and validation

The total number of SNP instruments for various dietary factors and the number of SNPs for each exposure are as follows: 35 SNPs for Alcoholic Drinks Per Week, 99 SNPs for Alcohol Intake Frequency, 41 SNPs for Tea Intake, 40 SNPs for Coffee Intake, 23 SNPs for Processed Meat Intake, 17 SNPs for Beef Intake, 8 SNPs for Poultry Intake, 11 SNPs for Non-Oily Fish Intake, 63 SNPs for Oily Fish Intake, 14 SNPs for Pork Intake, 17 SNPs for Cooked Vegetable Intake, 22 SNPs for Salad/Raw Vegetable Intake, 43 SNPs for Dried Fruit Intake, 43 SNPs for Cereal Intake, 32 SNPs for Bread Intake, and 65 SNPs for Cheese Intake. It’s noteworthy that the F-statistics of each SNP exceeded 10, indicating that the selected SNPs were strongly associated with the respective dietary exposures.

The number of individuals of European descent in the exposure datasets varied, ranging from 335,394 to 468,860 individuals. For the hypertension outcome, the study involved 92,462 cases of essential (primary) hypertension and 265,626 control subjects, which were sourced from the FinnGen Study.

To fortify the robustness of our analysis, we meticulously excluded outliers pinpointed by the MR-PRESSO methodology and SNPs that exhibited potential associations with confounding risk variables. Consequently, the spectrum of SNPs in our analysis varied, ranging from 7 to 85 across distinct dietary exposures. This refined selection strategy augmented the integrity and precision of our evaluation concerning the causal links between dietary components and the propensity for hypertension. Comprehensive insights into the specific exposures examined and corresponding outcomes can be gleaned from Table 1.

**Table 1:** Overview of the data sources of the IVs used in the MR study

IEU GWAS id	Exposure/outcome	SNPs/Used SNPs	Sample Size	Ancestry	PMID/Consortia	Source
ieu-b-73	Alcoholic drinks per week	35/30	335,394	European	30,643,251	NA
ukb-b-5779	Alcohol intake frequency	99/85	462,346	European	UKBiobank	https://biobank.ctsu.ox.ac.uk/crystal/field.cgi?id=1558
ukb-b-6066	Tea intake	41/36	447,485	European	UKBiobank	https://biobank.ctsu.ox.ac.uk/crystal/field.cgi?id=1488
ukb-b-5237	Coffee intake	40/32	428,860	European	UKBiobank	https://biobank.ctsu.ox.ac.uk/crystal/field.cgi?id=1498
ukb-b-6324	Processed meat intake	23/18	461,981	European	UKBiobank	https://biobank.ctsu.ox.ac.uk/crystal/field.cgi?id=1349
ukb-b-2862	Beef intake	17/10	461,053	European	UKBiobank	https://biobank.ctsu.ox.ac.uk/crystal/field.cgi?id=1369
ukb-b-8006	Poultry intake	8/7	461,900	European	UKBiobank	https://biobank.ctsu.ox.ac.uk/crystal/field.cgi?id=1359
ukb-b-17,627	Non-oily fish intake	11/10	460,880	European	UKBiobank	https://biobank.ctsu.ox.ac.uk/crystal/field.cgi?id=1339
ukb-b-2209	Oily fish intake	63/52	460,443	European	UKBiobank	https://biobank.ctsu.ox.ac.uk/crystal/field.cgi?id=1329
ukb-b-5640	Pork intake	14/13	460,162	European	UKBiobank	https://biobank.ctsu.ox.ac.uk/crystal/field.cgi?id=1389
ukb-b-8089	Cooked vegetable intake	17/14	448,651	European	UKBiobank	https://biobank.ctsu.ox.ac.uk/crystal/field.cgi?id=1289
ukb-b-1996	Salad / raw vegetable intake	22/18	435,435	European	UKBiobank	https://biobank.ctsu.ox.ac.uk/crystal/field.cgi?id=1299
ukb-b-16,576	Dried fruit intake	43/31	421,764	European	UKBiobank	https://biobank.ctsu.ox.ac.uk/crystal/field.cgi?id=1319
ukb-b-15,926	Cereal intake	43/30	441,640	European	UKBiobank	https://biobank.ctsu.ox.ac.uk/crystal/field.cgi?id=1458
ukb-b-11,348	Bread intake	32/22	452,236	European	UKBiobank	https://biobank.ctsu.ox.ac.uk/crystal/field.cgi?id=1438
ukb-b-1489	Cheese intake	65/51	451,486	European	UKBiobank	https://biobank.ctsu.ox.ac.uk/crystal/field.cgi?id=1408
Finn-b-I9_hypertension	Hypertension	NA	95,462 cases/ 265,626 controls	European	FINNGEN	https://r9.finngen.fi/

Abbreviations: MR: Mendelian randomization; IVs: instrumental variants; SNP: single nucleotide polymorphisms

### The causal role of dietary factors and hypertension

Our analysis pinpointed eight salient causal relationships, each demonstrating statistical significance at a threshold of *P* < 0.05, as ascertained through the IVW methodology. Specifically, findings illuminated that variables such as weekly alcohol consumption (OR: 1.53, 95% CI: 1.19–1.96), frequency of alcohol intake (OR: 1.20, 95% CI: 1.08–1.33), poultry consumption (OR: 3.25, 95% CI: 1.83–5.78), and beef consumption (OR: 1.80, 95% CI: 1.09–2.97) correlated positively with hypertension risk. In contrast, protective attributes were discerned for factors like raw salad/vegetable consumption (OR: 0.58, 95% CI: 0.37–0.90), dried fruit consumption (OR: 0.51, 95% CI: 0.37–0.70), cheese consumption (OR: 0.62, 95% CI: 0.51–0.75), and cereal consumption (OR: 0.65, 95% CI: 0.49–0.94).

Further evaluations indicated that certain factors, including processed meat intake (OR: 0.79, 95% CI: 0.60–1.04) and tea consumption (OR: 0.86, 95% CI: 0.73–1.02), did not manifest a statistically significant causal association with hypertension.

To bolster the credibility and robustness of our results, an array of analytical methodologies was employed to scrutinize both pleiotropy and heterogeneity. These encompassed the MR-Egger intercept test, leave-one-out analyses, Cochran’s Q test, and funnel plots. Our assessment revealed that the heterogeneity, as indicated by a Cochran Q-derived *P* value < 0.05, remained within acceptable limits when scrutinized through the random-effects IVW methodology [21]. Moreover, all MR-Egger intercept test *P*-values surpassed the 0.05 threshold, negating concerns of horizontal pleiotropy. For enhanced interpretability and visualization, an assortment of graphical representations—including scatter plots, forest plots, leave-one-out analyses, and funnel plots—has been encapsulated in Supplementary Figure S1-4, collectively reinforcing the observed associations between dietary variables and hypertension outcomes.

## Discussion

In this investigative endeavor, MR served as the cornerstone methodology to decipher the causal relationships between diverse dietary factors and susceptibility to hypertension. By leveraging genetically inferred dietary variables, this MR analysis demonstrated that genetic predisposition towards elevated consumption of alcoholic beverages per week, frequent alcohol ingestion, as well as increased poultry and beef consumption, were simultaneous with increased hypertension risk. Conversely, a genetic inclination towards augmented intake of cheese, cereal, raw vegetables, and dried fruits appeared to confer a protective effect against hypertension. Nevertheless, compelling evidence to establish a causative link between other dietary factors and hypertension remained elusive. The implications of the research may encourage hypertensive individuals to adopt a shift towards more balanced dietary regimes. For those at elevated hypertension risk, adhering to wholesome dietary practices may serve as a pivotal risk mitigation strategy.

In the current study, a genetic predisposition to higher alcohol consumption in terms of drinks per week and intake frequency was associated with an elevated risk of hypertension, consistent with previous observational studies and MR assumptions [2, 18]. Notably, Prior studies delineate a linear dose-response relationship between alcohol consumption and hypertension susceptibility in males, and a nonlinear trajectory has been discerned among females [19]. Whereas, recent meta-analyses have contravened erstwhile notions by asserting that light-to-moderate alcohol consumption among women fails to confer a protective mantle against hypertension [20, 21]. The cardioprotective role of light-to-moderate alcohol consumption remains shrouded in ambiguity due to limited evidence. Our study has demonstrated that the rs1229984 variant (Supplementary Table S1) was closely associated with alcohol consumption, which was also estimated in previous MR analyses [22]. The rs1229984 variant heightens aldehyde dehydrogenase 1 activity and leads to more rapid metabolisms of alcohol, furthermore, it might be related to the risk of hypertension. Therefore, the results have consistently demonstrated the relationship between alcohol intake and hypertension is causal. Crucially, it warrants acknowledgment that the scope of our MR analysis remained circumscribed, precluding an exhaustive exploration of nonlinear alcohol-hypertension associations; thus, our interpretations predominantly pivot on delineating the prospective causal ramifications of alcohol vis-à-vis hypertension susceptibility.

**Fig. 2:**
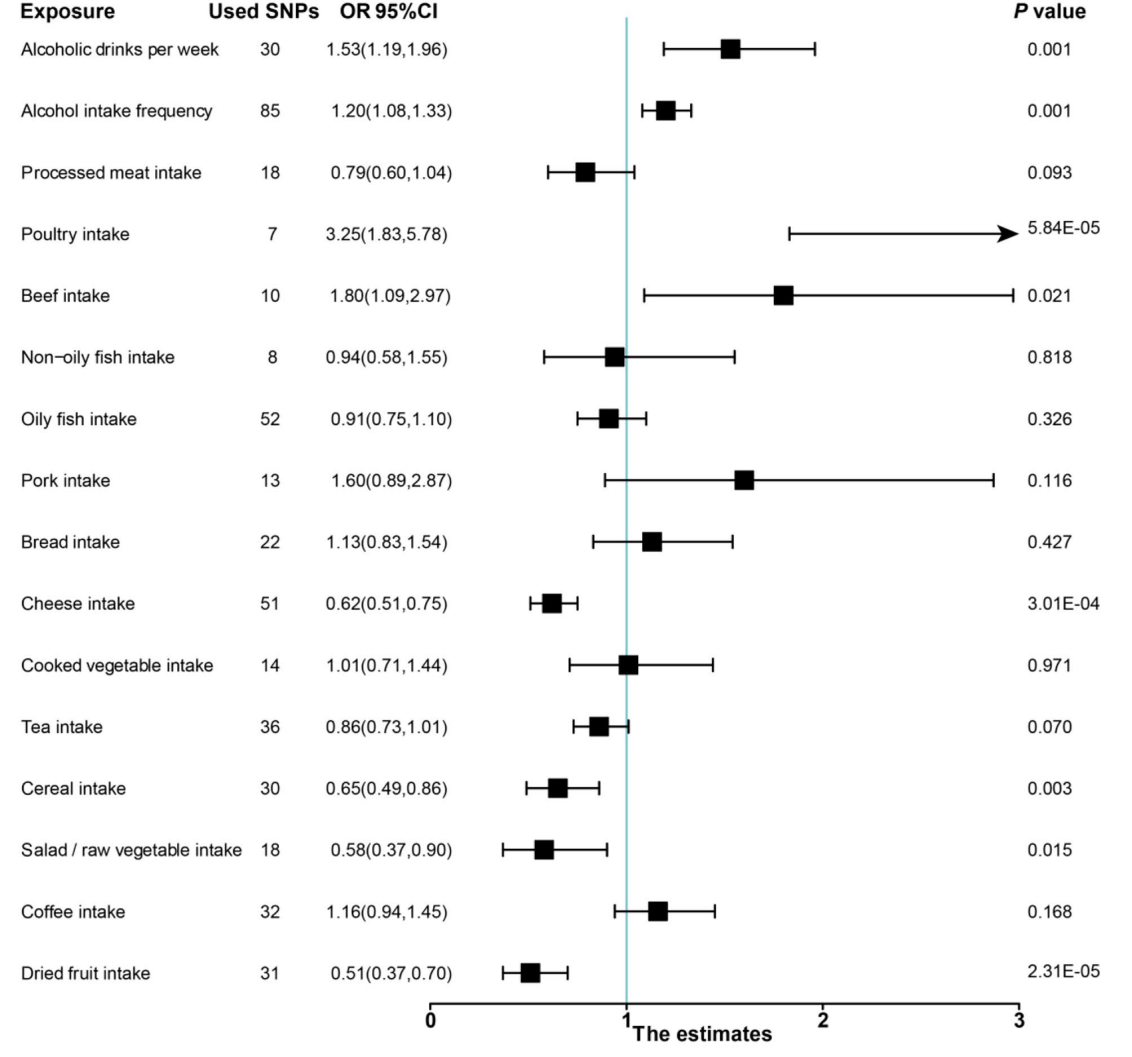
The association between dietary factors and hypertension using the IVW MR method; MR: Mendelian randomization; SNP: single nucleotide polymorphisms; IVW: inverse-variance weighted

Cohort studies investigating the association between meat intake and hypertension have produced inconsistent findings. Several studies have reported a positive link between red meat consumption, encompassing beef, lamb, and pork, and hypertension, conversely, inconsistent associations have been observed with poultry intake [23–25]. In our WM analysis, although the correlation between the consumption of poultry and hypertension was non-significant (Table 2), the IVW methodology showed a significant *P*-value, which confirmed the associations between poultry consumption and the risk of hypertension. Meanwhile, the impact of meat on the risk of hypertension may vary depending on the type. Lajous et al. found that consuming unprocessed red meat was not associated with hypertension [26], whereas, other studies had shown both processed and unprocessed meat were positively associated with elevated blood pressure [24]. Considering these discrepancies above, the effect of processed meats including sausages, cold cuts, and similar forms, on hypertension may be attributed to their sodium content [26, 27]. Moreover, the principal constituents of saturated fat and cholesterol in meat have also been demonstrated to be associated with hypertension [25]. Intriguingly, our findings found no association between processed meat and hypertension, potentially attributed to the U-shaped associations identified in the China Health and Nutrition Survey [28]. Therefore, there might be a non-linear association between meat consumption and hypertension risk, possibly manifesting as a U-shaped or J-shaped trajectory, which our study did not capture. Furthermore, our evidence accentuates a noteworthy insight: the consumption of red meat and poultry appears to concomitantly escalate the risk of hypertension, as manifestly elucidated by ORs eclipsing the unity threshold, as depicted in Fig. 2 [29]. This underscores an emergent mandate to judiciously regulate and modulate the consumption of these dietary constituents to mitigate hypertension risk profiles potentially.

**Table 2:** Associations between dietary factors and hypertension in analyses using the weighted-median and MR-Egger method

Exposure	Weighted median		MR-Egger		Cochrane’s test		Pleiotropy		
	OR (95% CI)	*P* value	OR (95% CI)	*P* value	Q value	*P* value	MR-Egger intercept	Se	*P* value
Alcoholic drinks per week	1.65(1.26,2.16)	7.18E-04	2.12(1.22,3.68)	0.013	62.71	0.001	-0.006	0.005	0.206
Alcohol intake frequency	1.14(1.01,1.29)	0.038	1.25(0.90,1.75)	0.191	198.73	2.72E-11	-0.001	0.004	0.780
Processed meat intake	0.87(0.62,1.24)	0.441	0.53(0.13,2.23)	0.399	23.55	0.132	0.006	0.011	0.583
Poultry intake	1.61E04(0,5.76E11)	0.325	2.55(1.26,5.15)	0.009	8.50	0.203	-0.092	0.096	0.382
Beef intake	1.74(0.97,3.13)	0.063	2.24(0.12,40.96)	0.602	13.29	0.150	-0.003	0.018	0.885
Non-oily fish intake	0.84(0.45,1.55)	0.571	1.29(0.09,18.08)	0.858	7.98	0.334	-0.004	0.016	0.822
Oily fish intake	0.79(0.63,1)	0.047	0.52(0.23,1.15)	0.113	91.01	0.001	0.008	0.006	0.163
Pork intake	2.09(0.05,93.99)	0.712	1.73(0.93,3.20)	0.081	27.16	0.001	-0.003	0.020	0.892
Bread intake	1.37(0.97,1.92)	0.070	1.55(0.39,6.10)	0.54	42.59	0.004	-0.004	0.01	0.653
Cheese intake	0.67(0.54,0.83)	1.76E-04	0.89(0.38,2.05)	0.777	104.01	1.16E-05	-0.006	0.007	0.396
Cooked vegetable intake	0.94(0.59,1.5)	0.804	0.15(0,7.67)	0.362	8.38	0.818	0.020	0.021	0.358
Tea intake	0.88(0.7,1.11)	0.283	0.92(0.63,1.34)	0.655	50.36	0.045	-0.001	0.003	0.704
Cereal intake	0.68(0.49,0.94)	0.018	0.45(0.15,1.41)	0.183	64.60	1.60E-04	0.005	0.009	0.525
Salad / raw vegetable intake	0.74(0.09,6.15)	0.788	0.78(0.45,1.35)	0.375	24.88	0.0975	-0.003	0.011	0.810
Coffee intake	0.95(0.73,1.23)	0.693	0.87(0.58,1.3)	0.503	55.81	0.004	0.006	0.003	0.104
Dried fruit intake	0.46(0.32,0.66)	2.36E-05	0.15(0.04,0.58)	0.010	64.92	2.24E-04	0.015	0.008	0.079

Abbreviations: MR: Mendelian randomization; IVs: instrumental variants; SNP: single nucleotide polymorphisms

Our study revealed that the consumption of salad/raw vegetables and dried fruits had a causal effect on reducing the risk of hypertension, aligning with previous observational studies [30–33]. Alissa et al. found that many constituents of fruit and vegetables (fiber, water-soluble vitamins, phytochemicals, and others) had multiple mechanisms, including reducing antioxidant stress, regulating hemostasis, and lowering blood pressure, thus, the intake of fruit and vegetables decreased the risk of hypertension [30, 31]. However, an unforeseen positive correlation between fruit consumption and hypertension among Vietnamese adults underscores the nuanced and potentially variable nature of the relationship between fruit intake and hypertension, particularly when scrutinized across diverse demographic cohorts [34]. This unpredicted association accentuates the imperative for further granular investigations to elucidate underlying mechanisms and contextual factors that might engender such divergent outcomes. Intriguingly, our MR scrutiny did not unearth any substantive association between the consumption of cooked vegetables and hypertension, potentially attributable to the elevated sodium content inherent in specific culinary preparations of vegetables.

Concurrently, our analyses unveiled an inverse causative nexus between cheese and cereal consumption and hypertension, a trend corroborated by prior cohort studies [35]. A corroborative two-sample MR analysis further bolstered the beneficial impact of cheese consumption on hypertension and associated cardiovascular disorders, including heart failure and coronary heart disease [36]. The plausible mechanistic underpinnings of these salubrious effects are multifaceted, encompassing cheese’s antioxidant and anti-inflammatory attributes in tandem with its rich reservoir of diverse minerals and proteins [36–38]. Previous studies have also suggested that cereal consumption might engender anti-hypertensive effects, augmenting endothelial or vascular functionality attributable to its ferulic acid constituents. Additionally, cereals have been ascribed an expansive repertoire of health-promoting attributes, spanning anti-inflammatory, anti-diabetic, anticancer, and cardioprotective dimensions [35, 39].

Therefore, the observed inverse associations between salad/raw vegetables, dried fruits, cheese, or cereals and hypertension may be causal, as they are consistent with findings from antecedent observational studies. Importantly, our results showed no discernible breaches of foundational assumptions, thereby amplifying the robustness and integrity of our empirically derived conclusions. In contrast, the null findings for processed meat, non-oily fish, oily fish, pork, bread, cooked vegetables, tea, and coffee consumption suggest that the associations observed in previous observational studies may be influenced by confounders or reverse causation bias [24, 40–42]. Notably, these null findings are particularly relevant for coffee consumption, as they lack strong biological plausibility for a causal association with hypertension [2]. While MR provides powerful and robust supplementary evidence to randomized controlled trials (RCTs), it should not be considered a substitute for RCTs. Therefore, these conclusions should be interpreted cautiously, and further research is needed to fully explore the causal relationships between these dietary factors and hypertension.

### Strengths and limitations

This analysis study has several strengths and limitations. Firstly, the employment of MR techniques leveraging genetic variations as IVs furnishes a robust bulwark against the pernicious influences of reverse causality and confounding variables, thereby fortifying the integrity of our findings [13, 43]. Secondly, to further enhance the fidelity and coherence of our MR evaluations, we judiciously incorporated MR-Egger and assorted pleiotropic analyses. Furthermore, the judicious amalgamation of exposure and outcome data harvested from diverse European cohorts ameliorates potential biases emanating from population-specific idiosyncrasies. However, the study has several limitations. Primarily, while the F-statistics attributable to the IVs underpinning our analysis exceeded the conventional threshold of 10, the majority hovered below the more stringent benchmark of 100, potentially attenuating result precision and fidelity. Secondly, the inherent constraints of our MR framework constrained our capacity to elucidate potentially nonlinear associations. Prevailing literature alludes to the potential existence of U- or J-shaped relationships in contexts like alcohol or red meat consumption vis-à-vis hypertension, wherein individuals manifesting light-to-moderate consumption trajectories ostensibly exhibit diminished hypertension risks relative to their non- or heavy-consuming counterparts. Consequently, adopting methodologies predicated on individual-level MR analyses could furnish nuanced insights into these intricate dynamics. Lastly, while attenuating the perils of population stratification biases, the ostensibly Eurocentric orientation of our participant cohort concurrently circumscribes the external validity and generalizability of our findings to more ethnically heterogeneous populations. This demographic skew warrants cautious interpretation and underscores the imperativeness of corroborative research endeavors across diverse ethnic cohorts to extrapolate and refine our understanding comprehensively.

## Conclusions

Our MR investigation has revealed pivotal insights into the intricate interplay between dietary factors and hypertension risk. Our findings elucidate that heightened consumption of alcoholic beverages, frequent alcohol ingestion, as well as increased poultry and beef consumption portend an augmented hypertension risk. However, a salubrious trend was discerned wherein increased consumption of raw vegetables, dried fruits, cheese, and cereals exhibited a protective, inverse causative association vis-à-vis hypertension. These findings provide valuable insights into the causal role of dietary factors in the prevention and management of hypertension.

### Electronic supplementary material

Below is the link to the electronic supplementary material.

Supplementary Material 1

## Data Availability

Publicly available datasets were utilized in this study. This data can be found here: the dietary factors GWAS data used were available in the IEU open GWAS project (https://gwas.mrcieu.ac.uk/) and the hypertension data applied could be found in the FinnGen Study (https://www.finngen.fi/fi).
