# Supplementary material for: Dietary factors in relation to hypertension: a mendelian randomization study
Source: J Health Popul Nutr. 2024 Jun 21;43:91. doi: 10.1186/s41043-024-00575-7 (PMC11193250; doi:10.1186/s41043-024-00575-7)
Supplement: Supplementary file 1 — Supplementary Material 1 [file 41043_2024_575_MOESM1_ESM.doc]

Table S1. Summary information for SNPs used as genetic instruments for MR analyses

| Exposure | Exposure ID | SNP | Effect allele exposure | Other allele exposure | Eaf | P value | F stastics |
| --- | --- | --- | --- | --- | --- | --- | --- |
| Alcoholic drinks per week | ieu-b-73 | rs28680958 | A | G | 0.23 | 9.78E-09 | 32.9 |
| Alcoholic drinks per week | ieu-b-73 | rs1260326 | C | T | 0.6 | 3.33E-33 | 144 |
| Alcoholic drinks per week | ieu-b-73 | rs6739804 | C | T | 0.66 | 4.72E-10 | 38.8 |
| Alcoholic drinks per week | ieu-b-73 | rs75120545 | T | C | 0.02 | 7.59E-09 | 33.4 |
| Alcoholic drinks per week | ieu-b-73 | rs71404478 | T | C | 0.34 | 3.83E-10 | 39.2 |
| Alcoholic drinks per week | ieu-b-73 | rs494904 | C | T | 0.43 | 1.41E-14 | 59.2 |
| Alcoholic drinks per week | ieu-b-73 | rs9835772 | T | A | 0.23 | 3.90E-09 | 34.7 |
| Alcoholic drinks per week | ieu-b-73 | rs28732378 | G | A | 0.73 | 2.24E-14 | 58.3 |
| Alcoholic drinks per week | ieu-b-73 | rs16854020 | A | G | 0.13 | 4.82E-10 | 38.7 |
| Alcoholic drinks per week | ieu-b-73 | rs78234152 | A | G | 0.1 | 2.18E-19 | 81.1 |
| Alcoholic drinks per week | ieu-b-73 | rs331939 | A | G | 0.34 | 4.50E-09 | 34.4 |
| Alcoholic drinks per week | ieu-b-73 | rs1229984 | C | T | 0.95 | 1.00E-200 | 926.99 |
| Alcoholic drinks per week | ieu-b-73 | rs13107325 | T | C | 0.07 | 1.23E-20 | 86.8 |
| Alcoholic drinks per week | ieu-b-73 | rs28712821 | A | G | 0.59 | 1.10E-46 | 206 |
| Alcoholic drinks per week | ieu-b-73 | rs55872084 | T | G | 0.22 | 1.98E-08 | 31.5 |
| Alcoholic drinks per week | ieu-b-73 | rs6969458 | A | G | 0.46 | 5.20E-11 | 43.1 |
| Alcoholic drinks per week | ieu-b-73 | rs2299409 | A | G | 0.49 | 4.80E-08 | 29.8 |
| Alcoholic drinks per week | ieu-b-73 | rs10085696 | G | A | 0.2 | 1.24E-10 | 41.4 |
| Alcoholic drinks per week | ieu-b-73 | rs28601761 | G | C | 0.41 | 7.60E-09 | 33.4 |
| Alcoholic drinks per week | ieu-b-73 | rs55932213 | G | A | 0.7 | 1.80E-08 | 31.7 |
| Alcoholic drinks per week | ieu-b-73 | rs4309187 | C | A | 0.7 | 1.37E-12 | 50.2 |
| Alcoholic drinks per week | ieu-b-73 | rs17542254 | G | A | 0.25 | 8.96E-10 | 37.5 |
| Alcoholic drinks per week | ieu-b-73 | rs2049045 | C | G | 0.19 | 3.97E-08 | 30.2 |
| Alcoholic drinks per week | ieu-b-73 | rs4752999 | T | C | 0.32 | 2.03E-12 | 49.5 |
| Alcoholic drinks per week | ieu-b-73 | rs1387766 | A | G | 0.62 | 4.79E-08 | 29.8 |
| Alcoholic drinks per week | ieu-b-73 | rs28929474 | T | C | 0.02 | 2.39E-11 | 44.6 |
| Alcoholic drinks per week | ieu-b-73 | rs962961 | T | C | 0.33 | 2.78E-09 | 35.3 |
| Alcoholic drinks per week | ieu-b-73 | rs11860773 | C | T | 0.18 | 8.35E-10 | 37.7 |
| Alcoholic drinks per week | ieu-b-73 | rs153106 | C | T | 0.41 | 3.63E-12 | 48.3 |
| Alcoholic drinks per week | ieu-b-73 | rs13332432 | G | C | 0.3 | 5.94E-11 | 42.8 |
| Alcoholic drinks per week | ieu-b-73 | rs79616692 | C | G | 0.11 | 2.38E-09 | 35.6 |
| Alcoholic drinks per week | ieu-b-73 | rs76640332 | A | G | 0.2 | 1.47E-18 | 77.3 |
| Alcoholic drinks per week | ieu-b-73 | rs34121753 | G | A | 0.53 | 1.39E-08 | 32.2 |
| Alcoholic drinks per week | ieu-b-73 | rs676388 | C | T | 0.49 | 5.49E-15 | 61.1 |
| Alcoholic drinks per week | ieu-b-73 | rs6106989 | A | G | 0.63 | 3.81E-08 | 30.2 |
| Alcohol intake frequency | ukb-b-5779 | rs780569 | A | T | 0.71 | 4.00E-09 | 34.64 |
| Alcohol intake frequency | ukb-b-5779 | rs4503294 | T | C | 0.57 | 3.40E-09 | 34.93 |
| Alcohol intake frequency | ukb-b-5779 | rs28787109 | A | G | 0.4 | 7.70E-09 | 33.34 |
| Alcohol intake frequency | ukb-b-5779 | rs2244598 | C | T | 0.61 | 3.80E-09 | 34.72 |
| Alcohol intake frequency | ukb-b-5779 | rs4417025 | A | G | 0.36 | 2.70E-09 | 35.42 |
| Alcohol intake frequency | ukb-b-5779 | rs7514579 | C | A | 0.23 | 4.60E-08 | 29.88 |
| Alcohol intake frequency | ukb-b-5779 | rs2717063 | A | C | 0.59 | 4.00E-11 | 43.61 |
| Alcohol intake frequency | ukb-b-5779 | rs6727281 | T | C | 0.18 | 5.50E-10 | 38.5 |
| Alcohol intake frequency | ukb-b-5779 | rs780094 | C | T | 0.62 | 1.30E-60 | 269.71 |
| Alcohol intake frequency | ukb-b-5779 | rs13390019 | C | T | 0.13 | 4.30E-11 | 43.46 |
| Alcohol intake frequency | ukb-b-5779 | rs10188314 | T | C | 0.47 | 7.20E-11 | 42.48 |
| Alcohol intake frequency | ukb-b-5779 | rs4241258 | T | C | 0.14 | 1.30E-08 | 32.4 |
| Alcohol intake frequency | ukb-b-5779 | rs72769229 | T | A | 0.15 | 3.40E-08 | 30.46 |
| Alcohol intake frequency | ukb-b-5779 | rs17662759 | C | T | 0.09 | 3.40E-08 | 30.46 |
| Alcohol intake frequency | ukb-b-5779 | rs1991083 | T | C | 0.68 | 6.30E-12 | 47.24 |
| Alcohol intake frequency | ukb-b-5779 | rs473098 | T | C | 0.56 | 9.10E-13 | 51.03 |
| Alcohol intake frequency | ukb-b-5779 | rs9829192 | T | G | 0.44 | 2.80E-08 | 30.81 |
| Alcohol intake frequency | ukb-b-5779 | rs76082653 | T | C | 0.05 | 3.80E-12 | 48.21 |
| Alcohol intake frequency | ukb-b-5779 | rs262240 | T | C | 0.47 | 1.40E-08 | 32.15 |
| Alcohol intake frequency | ukb-b-5779 | rs9814516 | T | G | 0.24 | 1.60E-12 | 49.87 |
| Alcohol intake frequency | ukb-b-5779 | rs7610856 | A | C | 0.43 | 7.70E-15 | 60.42 |
| Alcohol intake frequency | ukb-b-5779 | rs1515591 | G | T | 0.38 | 4.90E-09 | 34.22 |
| Alcohol intake frequency | ukb-b-5779 | rs1228589 | A | G | 0.25 | 2.30E-09 | 35.67 |
| Alcohol intake frequency | ukb-b-5779 | rs28622224 | T | C | 0.28 | 3.20E-08 | 30.56 |
| Alcohol intake frequency | ukb-b-5779 | rs13135092 | G | A | 0.08 | 1.60E-15 | 63.54 |
| Alcohol intake frequency | ukb-b-5779 | rs11940694 | G | A | 0.6 | 1.00E-44 | 196.8 |
| Alcohol intake frequency | ukb-b-5779 | rs362307 | T | C | 0.07 | 8.40E-14 | 55.7 |
| Alcohol intake frequency | ukb-b-5779 | rs1229984 | C | T | 0.97 | 1.40E-178 | 811.85 |
| Alcohol intake frequency | ukb-b-5779 | rs13102973 | C | T | 0.62 | 4.90E-10 | 38.72 |
| Alcohol intake frequency | ukb-b-5779 | rs62339673 | A | C | 0.63 | 6.60E-09 | 33.64 |
| Alcohol intake frequency | ukb-b-5779 | rs34811474 | A | G | 0.23 | 1.90E-08 | 31.55 |
| Alcohol intake frequency | ukb-b-5779 | rs2159935 | A | G | 0.49 | 8.30E-10 | 37.68 |
| Alcohol intake frequency | ukb-b-5779 | rs62305780 | G | C | 0.1 | 9.90E-22 | 91.74 |
| Alcohol intake frequency | ukb-b-5779 | rs13178443 | T | C | 0.28 | 3.80E-08 | 30.27 |
| Alcohol intake frequency | ukb-b-5779 | rs11750777 | A | G | 0.21 | 3.80E-08 | 30.25 |
| Alcohol intake frequency | ukb-b-5779 | rs4916723 | C | A | 0.42 | 1.10E-14 | 59.7 |
| Alcohol intake frequency | ukb-b-5779 | rs461599 | C | A | 0.46 | 2.70E-10 | 39.85 |
| Alcohol intake frequency | ukb-b-5779 | rs56194430 | T | C | 0.17 | 3.10E-08 | 30.65 |
| Alcohol intake frequency | ukb-b-5779 | rs9403297 | A | G | 0.37 | 1.80E-09 | 36.16 |
| Alcohol intake frequency | ukb-b-5779 | rs9349379 | G | A | 0.41 | 3.50E-10 | 39.4 |
| Alcohol intake frequency | ukb-b-5779 | rs12153855 | C | T | 0.1 | 2.40E-09 | 35.6 |
| Alcohol intake frequency | ukb-b-5779 | rs9372625 | A | G | 0.38 | 2.90E-16 | 66.9 |
| Alcohol intake frequency | ukb-b-5779 | rs62466318 | T | C | 0.2 | 1.40E-11 | 45.62 |
| Alcohol intake frequency | ukb-b-5779 | rs2622167 | A | G | 0.43 | 4.60E-10 | 38.84 |
| Alcohol intake frequency | ukb-b-5779 | rs73050128 | A | C | 0.16 | 2.10E-10 | 40.41 |
| Alcohol intake frequency | ukb-b-5779 | rs6943160 | C | T | 0.21 | 3.10E-08 | 30.62 |
| Alcohol intake frequency | ukb-b-5779 | rs4726481 | T | G | 0.4 | 2.30E-12 | 49.22 |
| Alcohol intake frequency | ukb-b-5779 | rs9648478 | A | G | 0.51 | 2.60E-08 | 30.99 |
| Alcohol intake frequency | ukb-b-5779 | rs2160935 | T | C | 0.6 | 1.40E-09 | 36.67 |
| Alcohol intake frequency | ukb-b-5779 | rs34440851 | T | C | 0.16 | 4.60E-08 | 29.87 |
| Alcohol intake frequency | ukb-b-5779 | rs11787216 | T | C | 0.37 | 2.40E-14 | 58.19 |
| Alcohol intake frequency | ukb-b-5779 | rs2977454 | G | C | 0.12 | 1.70E-08 | 31.78 |
| Alcohol intake frequency | ukb-b-5779 | rs74679146 | C | T | 0.07 | 2.50E-08 | 31.03 |
| Alcohol intake frequency | ukb-b-5779 | rs489062 | A | G | 0.44 | 4.90E-08 | 29.74 |
| Alcohol intake frequency | ukb-b-5779 | rs34473884 | A | G | 0.25 | 6.20E-09 | 33.78 |
| Alcohol intake frequency | ukb-b-5779 | rs61873510 | T | G | 0.33 | 6.90E-10 | 38.04 |
| Alcohol intake frequency | ukb-b-5779 | rs4242715 | A | G | 0.68 | 9.30E-09 | 32.98 |
| Alcohol intake frequency | ukb-b-5779 | rs10792669 | G | A | 0.51 | 9.90E-09 | 32.87 |
| Alcohol intake frequency | ukb-b-5779 | rs11223617 | A | G | 0.21 | 2.30E-11 | 44.68 |
| Alcohol intake frequency | ukb-b-5779 | rs550942 | T | C | 0.82 | 2.00E-08 | 31.54 |
| Alcohol intake frequency | ukb-b-5779 | rs11039429 | T | C | 0.45 | 8.70E-15 | 60.16 |
| Alcohol intake frequency | ukb-b-5779 | rs1666658 | C | T | 0.39 | 6.70E-09 | 33.62 |
| Alcohol intake frequency | ukb-b-5779 | rs12312693 | C | T | 0.45 | 6.80E-09 | 33.6 |
| Alcohol intake frequency | ukb-b-5779 | rs7302200 | A | G | 0.34 | 8.40E-09 | 33.18 |
| Alcohol intake frequency | ukb-b-5779 | rs28768122 | C | T | 0.76 | 5.60E-09 | 33.96 |
| Alcohol intake frequency | ukb-b-5779 | rs7298932 | G | A | 0.15 | 3.80E-08 | 30.27 |
| Alcohol intake frequency | ukb-b-5779 | rs58905411 | A | G | 0.41 | 5.10E-18 | 74.85 |
| Alcohol intake frequency | ukb-b-5779 | rs1937522 | G | A | 0.53 | 2.50E-08 | 31.06 |
| Alcohol intake frequency | ukb-b-5779 | rs7330939 | T | C | 0.72 | 3.70E-10 | 39.25 |
| Alcohol intake frequency | ukb-b-5779 | rs2535911 | T | C | 0.35 | 2.70E-09 | 35.38 |
| Alcohol intake frequency | ukb-b-5779 | rs186347 | T | G | 0.46 | 4.00E-09 | 34.62 |
| Alcohol intake frequency | ukb-b-5779 | rs80292319 | C | T | 0.06 | 1.40E-09 | 36.74 |
| Alcohol intake frequency | ukb-b-5779 | rs117799466 | C | G | 0.34 | 3.10E-09 | 35.11 |
| Alcohol intake frequency | ukb-b-5779 | rs34631026 | T | C | 0.45 | 2.90E-08 | 30.78 |
| Alcohol intake frequency | ukb-b-5779 | rs72787062 | A | G | 0.16 | 6.40E-12 | 47.21 |
| Alcohol intake frequency | ukb-b-5779 | rs35105141 | T | C | 0.4 | 1.40E-17 | 72.79 |
| Alcohol intake frequency | ukb-b-5779 | rs1421085 | C | T | 0.4 | 1.00E-10 | 41.78 |
| Alcohol intake frequency | ukb-b-5779 | rs1104608 | C | G | 0.43 | 1.70E-08 | 31.82 |
| Alcohol intake frequency | ukb-b-5779 | rs8043563 | C | G | 0.74 | 1.70E-11 | 45.31 |
| Alcohol intake frequency | ukb-b-5779 | rs2411453 | G | T | 0.6 | 7.30E-30 | 128.85 |
| Alcohol intake frequency | ukb-b-5779 | rs728538 | G | T | 0.17 | 1.80E-08 | 31.7 |
| Alcohol intake frequency | ukb-b-5779 | rs9906502 | A | G | 0.18 | 1.90E-09 | 36.05 |
| Alcohol intake frequency | ukb-b-5779 | rs8614 | A | C | 0.18 | 2.70E-10 | 39.85 |
| Alcohol intake frequency | ukb-b-5779 | rs4968391 | T | G | 0.67 | 2.30E-09 | 35.67 |
| Alcohol intake frequency | ukb-b-5779 | rs9912298 | C | A | 0.24 | 9.70E-09 | 32.89 |
| Alcohol intake frequency | ukb-b-5779 | rs17690703 | T | C | 0.26 | 2.90E-13 | 53.26 |
| Alcohol intake frequency | ukb-b-5779 | rs650558 | T | C | 0.25 | 3.40E-09 | 34.94 |
| Alcohol intake frequency | ukb-b-5779 | rs1893659 | A | C | 0.46 | 7.60E-22 | 92.26 |
| Alcohol intake frequency | ukb-b-5779 | rs5022348 | T | C | 0.41 | 1.40E-08 | 32.22 |
| Alcohol intake frequency | ukb-b-5779 | rs2043677 | T | C | 0.15 | 1.60E-09 | 36.42 |
| Alcohol intake frequency | ukb-b-5779 | rs9958320 | C | T | 0.15 | 5.90E-09 | 33.87 |
| Alcohol intake frequency | ukb-b-5779 | rs62097995 | A | T | 0.42 | 6.90E-11 | 42.54 |
| Alcohol intake frequency | ukb-b-5779 | rs2924321 | A | G | 0.54 | 1.60E-10 | 40.92 |
| Alcohol intake frequency | ukb-b-5779 | rs4940926 | C | T | 0.74 | 2.80E-08 | 30.82 |
| Alcohol intake frequency | ukb-b-5779 | rs838145 | A | G | 0.54 | 6.70E-13 | 51.63 |
| Alcohol intake frequency | ukb-b-5779 | rs6030200 | A | G | 0.31 | 2.40E-09 | 35.65 |
| Alcohol intake frequency | ukb-b-5779 | rs11700855 | G | A | 0.09 | 1.20E-08 | 32.42 |
| Alcohol intake frequency | ukb-b-5779 | rs71651683 | T | C | 0.01 | 3.60E-08 | 30.35 |
| Alcohol intake frequency | ukb-b-5779 | rs1894544 | C | G | 0.45 | 1.10E-08 | 32.6 |
| Processed meat intake | ukb-b-6324 | rs7531118 | C | T | 0.53 | 2.80E-11 | 44.33 |
| Processed meat intake | ukb-b-6324 | rs77165542 | T | C | 0.04 | 3.30E-09 | 35.02 |
| Processed meat intake | ukb-b-6324 | rs11887120 | T | C | 0.4 | 3.10E-08 | 30.66 |
| Processed meat intake | ukb-b-6324 | rs11894162 | T | C | 0.55 | 1.10E-08 | 32.7 |
| Processed meat intake | ukb-b-6324 | rs4077924 | C | T | 0.7 | 4.50E-08 | 29.93 |
| Processed meat intake | ukb-b-6324 | rs3762621 | T | C | 0.18 | 3.60E-08 | 30.38 |
| Processed meat intake | ukb-b-6324 | rs9809856 | G | A | 0.48 | 2.50E-10 | 40.06 |
| Processed meat intake | ukb-b-6324 | rs2873054 | C | A | 0.35 | 1.60E-10 | 40.85 |
| Processed meat intake | ukb-b-6324 | rs6786550 | C | T | 0.64 | 2.10E-08 | 31.42 |
| Processed meat intake | ukb-b-6324 | rs6765179 | A | G | 0.31 | 1.80E-08 | 31.71 |
| Processed meat intake | ukb-b-6324 | rs10454812 | C | A | 0.1 | 6.70E-09 | 33.63 |
| Processed meat intake | ukb-b-6324 | rs2029401 | G | A | 0.59 | 6.30E-12 | 47.22 |
| Processed meat intake | ukb-b-6324 | rs1422192 | A | G | 0.16 | 3.40E-09 | 34.92 |
| Processed meat intake | ukb-b-6324 | rs6961970 | A | C | 0.24 | 9.50E-09 | 32.95 |
| Processed meat intake | ukb-b-6324 | rs4240672 | A | G | 0.49 | 3.00E-16 | 66.84 |
| Processed meat intake | ukb-b-6324 | rs6484504 | C | T | 0.72 | 4.40E-11 | 43.43 |
| Processed meat intake | ukb-b-6324 | rs11032380 | T | A | 0.33 | 2.10E-09 | 35.92 |
| Processed meat intake | ukb-b-6324 | rs4778053 | G | C | 0.84 | 1.30E-08 | 32.27 |
| Processed meat intake | ukb-b-6324 | rs34241936 | G | A | 0.04 | 1.10E-08 | 32.57 |
| Processed meat intake | ukb-b-6324 | rs8096167 | C | T | 0.19 | 4.70E-08 | 29.86 |
| Processed meat intake | ukb-b-6324 | rs838133 | G | A | 0.55 | 1.60E-18 | 77.09 |
| Processed meat intake | ukb-b-6324 | rs6010651 | C | A | 0.38 | 1.10E-08 | 32.75 |
| Processed meat intake | ukb-b-6324 | rs203319 | T | C | 0.2 | 2.80E-10 | 39.84 |
| Poultry intake | ukb-b-8006 | rs9997448 | T | C | 0.37 | 2.70E-08 | 30.89 |
| Poultry intake | ukb-b-8006 | rs7829800 | G | A | 0.67 | 3.70E-09 | 34.78 |
| Poultry intake | ukb-b-8006 | rs7046351 | A | T | 0.51 | 1.10E-09 | 37.13 |
| Poultry intake | ukb-b-8006 | rs1051730 | A | G | 0.33 | 1.70E-08 | 31.78 |
| Poultry intake | ukb-b-8006 | rs9923768 | A | G | 0.6 | 1.60E-08 | 31.98 |
| Poultry intake | ukb-b-8006 | rs2565017 | A | G | 0.37 | 5.90E-09 | 33.86 |
| Poultry intake | ukb-b-8006 | rs2965200 | A | G | 0.64 | 4.20E-08 | 30.05 |
| Poultry intake | ukb-b-8006 | rs2426440 | G | A | 0.73 | 4.70E-08 | 29.85 |
| Beef intake | ukb-b-2862 | rs11165829 | G | C | 0.36 | 9.80E-09 | 32.89 |
| Beef intake | ukb-b-2862 | rs1105388 | T | C | 0.3 | 1.30E-09 | 36.78 |
| Beef intake | ukb-b-2862 | rs10789340 | G | A | 0.63 | 6.80E-15 | 60.65 |
| Beef intake | ukb-b-2862 | rs1470610 | C | G | 0.2 | 1.50E-08 | 32.05 |
| Beef intake | ukb-b-2862 | rs62169335 | T | C | 0.54 | 2.40E-08 | 31.16 |
| Beef intake | ukb-b-2862 | rs4676964 | T | C | 0.51 | 9.60E-15 | 59.97 |
| Beef intake | ukb-b-2862 | rs62396185 | C | G | 0.26 | 2.80E-14 | 57.89 |
| Beef intake | ukb-b-2862 | rs7791463 | A | G | 0.53 | 2.40E-08 | 31.16 |
| Beef intake | ukb-b-2862 | rs79809011 | A | G | 0.03 | 3.40E-08 | 30.45 |
| Beef intake | ukb-b-2862 | rs9407624 | A | T | 0.49 | 1.10E-15 | 64.33 |
| Beef intake | ukb-b-2862 | rs10959890 | C | T | 0.21 | 1.50E-09 | 36.51 |
| Beef intake | ukb-b-2862 | rs12247907 | C | G | 0.49 | 8.90E-09 | 33.07 |
| Beef intake | ukb-b-2862 | rs1421085 | C | T | 0.4 | 3.50E-12 | 48.39 |
| Beef intake | ukb-b-2862 | rs784251 | T | C | 0.48 | 1.70E-09 | 36.28 |
| Beef intake | ukb-b-2862 | rs429358 | C | T | 0.15 | 3.60E-10 | 39.32 |
| Beef intake | ukb-b-2862 | rs11878917 | A | G | 0.11 | 4.60E-08 | 29.87 |
| Beef intake | ukb-b-2862 | rs132901 | T | C | 0.79 | 2.90E-11 | 44.27 |
| Non-oily fish intake | ukb-b-17627 | rs16822430 | C | T | 0.23 | 1.40E-09 | 36.72 |
| Non-oily fish intake | ukb-b-17627 | rs1260326 | C | T | 0.6 | 7.90E-09 | 33.31 |
| Non-oily fish intake | ukb-b-17627 | rs11680516 | C | T | 0.2 | 1.40E-09 | 36.68 |
| Non-oily fish intake | ukb-b-17627 | rs3799077 | G | T | 0.31 | 1.00E-09 | 37.3 |
| Non-oily fish intake | ukb-b-17627 | rs4318925 | T | C | 0.18 | 1.30E-12 | 50.28 |
| Non-oily fish intake | ukb-b-17627 | rs6957745 | C | T | 0.2 | 1.80E-09 | 36.23 |
| Non-oily fish intake | ukb-b-17627 | rs17317920 | G | A | 0.48 | 2.80E-08 | 30.85 |
| Non-oily fish intake | ukb-b-17627 | rs35287743 | T | G | 0.12 | 3.60E-12 | 48.31 |
| Non-oily fish intake | ukb-b-17627 | rs7148387 | G | A | 0.59 | 1.70E-08 | 31.8 |
| Non-oily fish intake | ukb-b-17627 | rs56094641 | G | A | 0.4 | 2.50E-14 | 58.12 |
| Non-oily fish intake | ukb-b-17627 | rs838133 | G | A | 0.55 | 4.70E-22 | 93.22 |
| Oily fish intake | ukb-b-2209 | rs973526 | T | C | 0.51 | 2.50E-09 | 35.56 |
| Oily fish intake | ukb-b-2209 | rs45501495 | T | C | 0.24 | 3.70E-12 | 48.26 |
| Oily fish intake | ukb-b-2209 | rs55930451 | T | C | 0.11 | 2.90E-08 | 30.74 |
| Oily fish intake | ukb-b-2209 | rs55985303 | A | G | 0.24 | 6.60E-09 | 33.66 |
| Oily fish intake | ukb-b-2209 | rs17050031 | T | C | 0.48 | 3.50E-10 | 39.35 |
| Oily fish intake | ukb-b-2209 | rs275160 | C | T | 0.7 | 8.00E-09 | 33.27 |
| Oily fish intake | ukb-b-2209 | rs13070166 | A | T | 0.23 | 4.40E-10 | 38.91 |
| Oily fish intake | ukb-b-2209 | rs114497213 | T | G | 0.05 | 1.10E-10 | 41.6 |
| Oily fish intake | ukb-b-2209 | rs10513136 | A | G | 0.07 | 1.60E-09 | 36.45 |
| Oily fish intake | ukb-b-2209 | rs1876245 | C | T | 0.43 | 5.00E-15 | 61.27 |
| Oily fish intake | ukb-b-2209 | rs10510554 | C | T | 0.57 | 1.20E-08 | 32.53 |
| Oily fish intake | ukb-b-2209 | rs905575 | G | C | 0.82 | 3.60E-08 | 30.37 |
| Oily fish intake | ukb-b-2209 | rs9841174 | C | T | 0.37 | 8.50E-14 | 55.69 |
| Oily fish intake | ukb-b-2209 | rs1201289 | G | T | 0.39 | 4.40E-08 | 29.97 |
| Oily fish intake | ukb-b-2209 | rs7683782 | G | C | 0.83 | 1.90E-08 | 31.64 |
| Oily fish intake | ukb-b-2209 | rs10076975 | C | T | 0.38 | 1.10E-08 | 32.64 |
| Oily fish intake | ukb-b-2209 | rs10061973 | T | G | 0.51 | 1.50E-08 | 32.08 |
| Oily fish intake | ukb-b-2209 | rs16891727 | A | C | 0.13 | 6.80E-17 | 69.74 |
| Oily fish intake | ukb-b-2209 | rs34555420 | T | G | 0.1 | 1.50E-13 | 54.51 |
| Oily fish intake | ukb-b-2209 | rs12663865 | A | G | 0.76 | 1.10E-08 | 32.73 |
| Oily fish intake | ukb-b-2209 | rs4869859 | C | T | 0.45 | 3.10E-13 | 53.13 |
| Oily fish intake | ukb-b-2209 | rs11767283 | G | A | 0.22 | 2.50E-14 | 58.06 |
| Oily fish intake | ukb-b-2209 | rs6465487 | G | A | 0.4 | 2.70E-10 | 39.88 |
| Oily fish intake | ukb-b-2209 | rs11986122 | G | C | 0.42 | 2.90E-14 | 57.78 |
| Oily fish intake | ukb-b-2209 | rs790564 | C | A | 0.72 | 7.90E-12 | 46.79 |
| Oily fish intake | ukb-b-2209 | rs552234 | A | G | 0.5 | 1.10E-09 | 37.09 |
| Oily fish intake | ukb-b-2209 | rs9886779 | A | T | 0.44 | 2.70E-08 | 30.89 |
| Oily fish intake | ukb-b-2209 | rs10828250 | G | C | 0.31 | 2.60E-22 | 94.41 |
| Oily fish intake | ukb-b-2209 | rs703987 | C | G | 0.62 | 1.70E-08 | 31.76 |
| Oily fish intake | ukb-b-2209 | rs61882686 | A | C | 0.09 | 8.00E-09 | 33.26 |
| Oily fish intake | ukb-b-2209 | rs4278546 | G | A | 0.44 | 9.30E-11 | 41.97 |
| Oily fish intake | ukb-b-2209 | rs2374424 | G | A | 0.6 | 4.90E-09 | 34.23 |
| Oily fish intake | ukb-b-2209 | rs510161 | G | C | 0.31 | 4.50E-08 | 29.91 |
| Oily fish intake | ukb-b-2209 | rs631490 | C | G | 0.71 | 6.00E-13 | 51.85 |
| Oily fish intake | ukb-b-2209 | rs303817 | G | A | 0.75 | 8.00E-10 | 37.77 |
| Oily fish intake | ukb-b-2209 | rs35287743 | T | G | 0.12 | 7.00E-21 | 87.86 |
| Oily fish intake | ukb-b-2209 | rs9597870 | G | T | 0.25 | 1.10E-08 | 32.65 |
| Oily fish intake | ukb-b-2209 | rs3124402 | G | A | 0.73 | 1.90E-24 | 104.09 |
| Oily fish intake | ukb-b-2209 | rs12855717 | T | C | 0.53 | 2.00E-10 | 40.46 |
| Oily fish intake | ukb-b-2209 | rs1361016 | G | T | 0.84 | 1.70E-08 | 31.78 |
| Oily fish intake | ukb-b-2209 | rs9301837 | A | C | 0.14 | 8.10E-09 | 33.24 |
| Oily fish intake | ukb-b-2209 | rs4982738 | A | G | 0.58 | 3.50E-08 | 30.41 |
| Oily fish intake | ukb-b-2209 | rs12896749 | C | G | 0.38 | 2.50E-08 | 31.1 |
| Oily fish intake | ukb-b-2209 | rs1951286 | G | T | 0.64 | 3.00E-13 | 53.2 |
| Oily fish intake | ukb-b-2209 | rs28533540 | A | G | 0.53 | 2.80E-14 | 57.87 |
| Oily fish intake | ukb-b-2209 | rs1421085 | C | T | 0.4 | 2.50E-21 | 89.94 |
| Oily fish intake | ukb-b-2209 | rs11859365 | C | A | 0.25 | 9.40E-25 | 105.53 |
| Oily fish intake | ukb-b-2209 | rs9889161 | T | G | 0.36 | 2.80E-11 | 44.29 |
| Oily fish intake | ukb-b-2209 | rs28623270 | T | A | 0.15 | 7.30E-11 | 42.44 |
| Oily fish intake | ukb-b-2209 | rs2952140 | T | C | 0.48 | 2.50E-08 | 31.09 |
| Oily fish intake | ukb-b-2209 | rs4510068 | T | G | 0.4 | 4.00E-11 | 43.6 |
| Oily fish intake | ukb-b-2209 | rs7243428 | G | A | 0.22 | 1.50E-08 | 32.06 |
| Oily fish intake | ukb-b-2209 | rs9958909 | G | T | 0.14 | 1.40E-08 | 32.19 |
| Oily fish intake | ukb-b-2209 | rs59355765 | T | C | 0.16 | 4.70E-10 | 38.78 |
| Oily fish intake | ukb-b-2209 | rs4002471 | T | C | 0.55 | 1.50E-23 | 100 |
| Oily fish intake | ukb-b-2209 | rs7254235 | G | A | 0.58 | 4.30E-08 | 30.02 |
| Oily fish intake | ukb-b-2209 | rs75887709 | G | A | 0.14 | 1.60E-08 | 31.95 |
| Oily fish intake | ukb-b-2209 | rs12983532 | T | C | 0.25 | 2.00E-09 | 35.94 |
| Oily fish intake | ukb-b-2209 | rs6033437 | A | C | 0.26 | 1.70E-08 | 31.84 |
| Oily fish intake | ukb-b-2209 | rs6059844 | G | A | 0.5 | 9.20E-09 | 33.01 |
| Oily fish intake | ukb-b-2209 | rs6089753 | T | C | 0.53 | 1.80E-09 | 36.2 |
| Oily fish intake | ukb-b-2209 | rs2827161 | G | T | 0.42 | 3.20E-08 | 30.57 |
| Oily fish intake | ukb-b-2209 | rs9606833 | C | T | 0.24 | 2.70E-14 | 57.97 |
| Pork intake | ukb-b-5640 | rs11211124 | C | T | 0.23 | 1.40E-08 | 32.19 |
| Pork intake | ukb-b-5640 | rs9973426 | G | A | 0.18 | 1.00E-08 | 32.76 |
| Pork intake | ukb-b-5640 | rs7641973 | A | G | 0.35 | 4.20E-08 | 30.04 |
| Pork intake | ukb-b-5640 | rs254152 | G | C | 0.23 | 2.20E-09 | 35.82 |
| Pork intake | ukb-b-5640 | rs9379832 | G | A | 0.26 | 1.80E-11 | 45.2 |
| Pork intake | ukb-b-5640 | rs10972033 | T | G | 0.46 | 1.30E-09 | 36.85 |
| Pork intake | ukb-b-5640 | rs1355171 | A | C | 0.49 | 1.00E-13 | 55.3 |
| Pork intake | ukb-b-5640 | rs34161520 | G | C | 0.16 | 9.60E-09 | 32.91 |
| Pork intake | ukb-b-5640 | rs2387807 | T | C | 0.08 | 4.10E-08 | 30.12 |
| Pork intake | ukb-b-5640 | rs4146837 | T | C | 0.46 | 4.00E-09 | 34.62 |
| Pork intake | ukb-b-5640 | rs3964074 | C | T | 0.55 | 1.60E-09 | 36.41 |
| Pork intake | ukb-b-5640 | rs36124222 | C | T | 0.43 | 2.10E-08 | 31.39 |
| Pork intake | ukb-b-5640 | rs12721051 | G | C | 0.19 | 5.60E-11 | 42.94 |
| Pork intake | ukb-b-5640 | rs838133 | G | A | 0.55 | 9.00E-13 | 51.04 |
| Bread intake | ukb-b-11348 | rs9662365 | T | C | 0.5 | 9.60E-10 | 37.41 |
| Bread intake | ukb-b-11348 | rs13023099 | A | C | 0.57 | 1.40E-08 | 32.16 |
| Bread intake | ukb-b-11348 | rs6754311 | C | T | 0.26 | 3.10E-10 | 39.59 |
| Bread intake | ukb-b-11348 | rs4665972 | C | T | 0.6 | 3.20E-12 | 48.54 |
| Bread intake | ukb-b-11348 | rs75287965 | A | G | 0.06 | 1.50E-09 | 36.48 |
| Bread intake | ukb-b-11348 | rs13016665 | A | C | 0.42 | 3.50E-13 | 52.92 |
| Bread intake | ukb-b-11348 | rs1492988 | G | C | 0.6 | 1.40E-08 | 32.17 |
| Bread intake | ukb-b-11348 | rs9832088 | A | T | 0.52 | 1.30E-13 | 54.84 |
| Bread intake | ukb-b-11348 | rs9881332 | G | C | 0.58 | 1.90E-08 | 31.62 |
| Bread intake | ukb-b-11348 | rs1994315 | C | T | 0.69 | 4.30E-15 | 61.55 |
| Bread intake | ukb-b-11348 | rs73802707 | T | C | 0.15 | 8.00E-09 | 33.27 |
| Bread intake | ukb-b-11348 | rs2068650 | C | A | 0.47 | 3.10E-12 | 48.6 |
| Bread intake | ukb-b-11348 | rs17083079 | A | G | 0.05 | 1.20E-10 | 41.41 |
| Bread intake | ukb-b-11348 | rs2517678 | T | C | 0.37 | 2.20E-10 | 40.24 |
| Bread intake | ukb-b-11348 | rs596878 | C | A | 0.45 | 5.30E-09 | 34.09 |
| Bread intake | ukb-b-11348 | rs79436018 | C | T | 0.12 | 1.60E-08 | 31.95 |
| Bread intake | ukb-b-11348 | rs7802468 | T | C | 0.37 | 6.90E-30 | 128.97 |
| Bread intake | ukb-b-11348 | rs10761661 | T | C | 0.45 | 1.00E-08 | 32.82 |
| Bread intake | ukb-b-11348 | rs55745436 | T | C | 0.24 | 1.00E-08 | 32.84 |
| Bread intake | ukb-b-11348 | rs1940033 | T | C | 0.59 | 4.70E-08 | 29.83 |
| Bread intake | ukb-b-11348 | rs11183201 | C | T | 0.51 | 5.30E-17 | 70.23 |
| Bread intake | ukb-b-11348 | rs6580721 | G | A | 0.19 | 1.30E-11 | 45.75 |
| Bread intake | ukb-b-11348 | rs11060853 | G | A | 0.41 | 2.50E-10 | 40.05 |
| Bread intake | ukb-b-11348 | rs9564268 | C | T | 0.62 | 3.00E-09 | 35.21 |
| Bread intake | ukb-b-11348 | rs9529024 | T | A | 0.37 | 4.20E-10 | 39.02 |
| Bread intake | ukb-b-11348 | rs11628639 | C | T | 0.24 | 6.20E-09 | 33.77 |
| Bread intake | ukb-b-11348 | rs9323989 | C | T | 0.38 | 1.60E-08 | 31.93 |
| Bread intake | ukb-b-11348 | rs28406095 | A | G | 0.46 | 4.40E-08 | 29.97 |
| Bread intake | ukb-b-11348 | rs4984685 | A | G | 0.2 | 4.40E-08 | 29.99 |
| Bread intake | ukb-b-11348 | rs62091167 | C | A | 0.22 | 1.20E-08 | 32.57 |
| Bread intake | ukb-b-11348 | rs656817 | G | A | 0.33 | 1.80E-09 | 36.19 |
| Bread intake | ukb-b-11348 | rs7276867 | C | G | 0.54 | 1.50E-08 | 32.1 |
| Cheese intake | ukb-b-1489 | rs78876700 | A | G | 0.14 | 3.40E-08 | 30.47 |
| Cheese intake | ukb-b-1489 | rs531358 | T | C | 0.65 | 1.80E-08 | 31.73 |
| Cheese intake | ukb-b-1489 | rs2802530 | A | G | 0.88 | 4.20E-08 | 30.07 |
| Cheese intake | ukb-b-1489 | rs6685323 | T | C | 0.31 | 4.80E-08 | 29.8 |
| Cheese intake | ukb-b-1489 | rs2339928 | A | G | 0.7 | 1.20E-09 | 36.93 |
| Cheese intake | ukb-b-1489 | rs12475594 | G | A | 0.18 | 4.40E-08 | 29.95 |
| Cheese intake | ukb-b-1489 | rs504675 | T | C | 0.35 | 1.00E-31 | 137.31 |
| Cheese intake | ukb-b-1489 | rs72970243 | A | G | 0.12 | 6.70E-11 | 42.6 |
| Cheese intake | ukb-b-1489 | rs1514755 | G | A | 0.24 | 3.90E-10 | 39.17 |
| Cheese intake | ukb-b-1489 | rs79184944 | A | T | 0.13 | 2.40E-09 | 35.61 |
| Cheese intake | ukb-b-1489 | rs4296548 | G | T | 0.61 | 1.20E-08 | 32.41 |
| Cheese intake | ukb-b-1489 | rs62245792 | A | T | 0.15 | 1.40E-08 | 32.14 |
| Cheese intake | ukb-b-1489 | rs77742462 | G | A | 0.02 | 9.80E-09 | 32.88 |
| Cheese intake | ukb-b-1489 | rs2352974 | T | C | 0.49 | 1.00E-10 | 41.78 |
| Cheese intake | ukb-b-1489 | rs6774906 | C | A | 0.04 | 2.50E-08 | 31.09 |
| Cheese intake | ukb-b-1489 | rs4681981 | A | C | 0.47 | 2.90E-08 | 30.79 |
| Cheese intake | ukb-b-1489 | rs4860341 | C | T | 0.93 | 2.20E-08 | 31.35 |
| Cheese intake | ukb-b-1489 | rs73096946 | C | T | 0.16 | 1.90E-11 | 45.09 |
| Cheese intake | ukb-b-1489 | rs13107325 | T | C | 0.07 | 7.00E-12 | 47.02 |
| Cheese intake | ukb-b-1489 | rs10938397 | G | A | 0.43 | 1.80E-08 | 31.68 |
| Cheese intake | ukb-b-1489 | rs4692708 | C | A | 0.25 | 1.30E-08 | 32.37 |
| Cheese intake | ukb-b-1489 | rs26579 | C | G | 0.59 | 2.40E-08 | 31.13 |
| Cheese intake | ukb-b-1489 | rs6873324 | C | A | 0.43 | 3.90E-08 | 30.19 |
| Cheese intake | ukb-b-1489 | rs9504123 | C | A | 0.27 | 1.50E-08 | 32.03 |
| Cheese intake | ukb-b-1489 | rs975303 | G | A | 0.18 | 2.50E-13 | 53.56 |
| Cheese intake | ukb-b-1489 | rs1931805 | C | T | 0.5 | 1.60E-08 | 31.94 |
| Cheese intake | ukb-b-1489 | rs113367286 | T | C | 0.28 | 1.30E-09 | 36.87 |
| Cheese intake | ukb-b-1489 | rs34198643 | T | C | 0.22 | 4.50E-10 | 38.87 |
| Cheese intake | ukb-b-1489 | rs12672200 | A | G | 0.33 | 9.00E-09 | 33.04 |
| Cheese intake | ukb-b-1489 | rs9649582 | T | A | 0.32 | 1.40E-09 | 36.74 |
| Cheese intake | ukb-b-1489 | rs7012814 | A | G | 0.47 | 2.10E-16 | 67.49 |
| Cheese intake | ukb-b-1489 | rs7386207 | T | C | 0.56 | 3.60E-08 | 30.33 |
| Cheese intake | ukb-b-1489 | rs13257887 | C | T | 0.36 | 2.70E-10 | 39.89 |
| Cheese intake | ukb-b-1489 | rs3911016 | G | T | 0.12 | 5.30E-10 | 38.55 |
| Cheese intake | ukb-b-1489 | rs4503172 | T | C | 0.61 | 1.60E-08 | 31.94 |
| Cheese intake | ukb-b-1489 | rs1806771 | G | T | 0.09 | 4.10E-08 | 30.13 |
| Cheese intake | ukb-b-1489 | rs73335955 | C | T | 0.05 | 2.40E-08 | 31.12 |
| Cheese intake | ukb-b-1489 | rs10896050 | T | G | 0.19 | 7.20E-11 | 42.46 |
| Cheese intake | ukb-b-1489 | rs67238148 | T | G | 0.22 | 1.10E-09 | 37.15 |
| Cheese intake | ukb-b-1489 | rs7936836 | A | C | 0.42 | 2.60E-12 | 48.96 |
| Cheese intake | ukb-b-1489 | rs73024305 | C | G | 0.05 | 4.00E-11 | 43.61 |
| Cheese intake | ukb-b-1489 | rs12786959 | T | A | 0.2 | 1.20E-08 | 32.48 |
| Cheese intake | ukb-b-1489 | rs524468 | G | A | 0.26 | 2.40E-08 | 31.15 |
| Cheese intake | ukb-b-1489 | rs1024853 | G | C | 0.44 | 1.30E-08 | 32.33 |
| Cheese intake | ukb-b-1489 | rs7298331 | C | A | 0.6 | 1.10E-08 | 32.65 |
| Cheese intake | ukb-b-1489 | rs12296440 | A | G | 0.17 | 2.80E-10 | 39.78 |
| Cheese intake | ukb-b-1489 | rs61953351 | T | G | 0.25 | 1.50E-08 | 31.99 |
| Cheese intake | ukb-b-1489 | rs1073242 | A | G | 0.55 | 6.70E-12 | 47.11 |
| Cheese intake | ukb-b-1489 | rs11620149 | C | T | 0.14 | 3.60E-08 | 30.35 |
| Cheese intake | ukb-b-1489 | rs17115145 | T | C | 0.4 | 1.80E-08 | 31.65 |
| Cheese intake | ukb-b-1489 | rs35270670 | G | A | 0.22 | 1.50E-09 | 36.54 |
| Cheese intake | ukb-b-1489 | rs4776970 | T | A | 0.36 | 3.50E-11 | 43.85 |
| Cheese intake | ukb-b-1489 | rs12447542 | A | G | 0.13 | 6.80E-09 | 33.6 |
| Cheese intake | ukb-b-1489 | rs61734410 | T | C | 0.26 | 2.20E-10 | 40.32 |
| Cheese intake | ukb-b-1489 | rs62034322 | A | G | 0.38 | 1.40E-09 | 36.71 |
| Cheese intake | ukb-b-1489 | rs71386942 | A | C | 0.27 | 9.90E-09 | 32.85 |
| Cheese intake | ukb-b-1489 | rs11649653 | G | C | 0.38 | 1.50E-09 | 36.52 |
| Cheese intake | ukb-b-1489 | rs919109 | C | G | 0.14 | 7.90E-10 | 37.79 |
| Cheese intake | ukb-b-1489 | rs2854175 | A | C | 0.26 | 3.70E-11 | 43.77 |
| Cheese intake | ukb-b-1489 | rs12951057 | G | C | 0.17 | 3.60E-12 | 48.31 |
| Cheese intake | ukb-b-1489 | rs2960578 | G | T | 0.5 | 2.60E-14 | 58 |
| Cheese intake | ukb-b-1489 | rs1434511 | T | C | 0.46 | 9.50E-09 | 32.95 |
| Cheese intake | ukb-b-1489 | rs1291145 | C | T | 0.69 | 4.40E-17 | 70.58 |
| Cheese intake | ukb-b-1489 | rs6126641 | A | G | 0.34 | 3.30E-08 | 30.5 |
| Cheese intake | ukb-b-1489 | rs62236533 | A | G | 0.11 | 1.10E-11 | 46.11 |
| Cooked vegetable intake | ukb-b-8089 | rs2252508 | G | A | 0.48 | 5.70E-09 | 33.95 |
| Cooked vegetable intake | ukb-b-8089 | rs2102738 | C | A | 0.17 | 5.30E-09 | 34.07 |
| Cooked vegetable intake | ukb-b-8089 | rs4851029 | G | T | 0.53 | 7.80E-11 | 42.31 |
| Cooked vegetable intake | ukb-b-8089 | rs12629972 | C | T | 0.59 | 1.20E-13 | 55.02 |
| Cooked vegetable intake | ukb-b-8089 | rs28450747 | A | G | 0.23 | 4.30E-08 | 30.01 |
| Cooked vegetable intake | ukb-b-8089 | rs1816263 | C | T | 0.28 | 3.70E-08 | 30.3 |
| Cooked vegetable intake | ukb-b-8089 | rs2844672 | A | G | 0.62 | 2.10E-09 | 35.9 |
| Cooked vegetable intake | ukb-b-8089 | rs12550717 | A | G | 0.37 | 1.40E-08 | 32.2 |
| Cooked vegetable intake | ukb-b-8089 | rs10156602 | G | A | 0.36 | 1.80E-11 | 45.21 |
| Cooked vegetable intake | ukb-b-8089 | rs11138705 | C | G | 0.76 | 1.40E-08 | 32.12 |
| Cooked vegetable intake | ukb-b-8089 | rs2052063 | T | C | 0.52 | 1.60E-09 | 36.38 |
| Cooked vegetable intake | ukb-b-8089 | rs349062 | C | G | 0.39 | 2.50E-08 | 31.06 |
| Cooked vegetable intake | ukb-b-8089 | rs28711392 | C | T | 0.37 | 4.60E-11 | 43.34 |
| Cooked vegetable intake | ukb-b-8089 | rs10161952 | C | A | 0.31 | 1.30E-08 | 32.29 |
| Cooked vegetable intake | ukb-b-8089 | rs1421085 | C | T | 0.4 | 8.30E-11 | 42.19 |
| Cooked vegetable intake | ukb-b-8089 | rs838133 | G | A | 0.55 | 4.50E-13 | 52.4 |
| Cooked vegetable intake | ukb-b-8089 | rs34155012 | T | C | 0.23 | 3.90E-08 | 30.18 |
| Tea intake | ukb-b-6066 | rs11587444 | G | A | 0.39 | 1.00E-10 | 41.79 |
| Tea intake | ukb-b-6066 | rs11164870 | G | C | 0.6 | 4.20E-08 | 30.04 |
| Tea intake | ukb-b-6066 | rs56188862 | C | T | 0.39 | 4.30E-13 | 52.5 |
| Tea intake | ukb-b-6066 | rs1156588 | G | A | 0.21 | 2.90E-09 | 35.24 |
| Tea intake | ukb-b-6066 | rs57462170 | A | G | 0.11 | 1.90E-08 | 31.62 |
| Tea intake | ukb-b-6066 | rs2117137 | G | A | 0.41 | 1.70E-09 | 36.34 |
| Tea intake | ukb-b-6066 | rs1481012 | G | A | 0.11 | 5.30E-15 | 61.15 |
| Tea intake | ukb-b-6066 | rs34619 | A | G | 0.43 | 4.30E-08 | 30.02 |
| Tea intake | ukb-b-6066 | rs72797284 | G | A | 0.27 | 7.00E-13 | 51.56 |
| Tea intake | ukb-b-6066 | rs7757102 | G | A | 0.56 | 3.10E-08 | 30.62 |
| Tea intake | ukb-b-6066 | rs2478875 | G | A | 0.21 | 5.10E-17 | 70.3 |
| Tea intake | ukb-b-6066 | rs149805207 | G | A | 0.01 | 1.10E-08 | 32.68 |
| Tea intake | ukb-b-6066 | rs4410790 | C | T | 0.63 | 3.40E-76 | 341.27 |
| Tea intake | ukb-b-6066 | rs17685 | A | G | 0.28 | 1.60E-22 | 95.36 |
| Tea intake | ukb-b-6066 | rs141071726 | A | G | 0.03 | 2.20E-09 | 35.75 |
| Tea intake | ukb-b-6066 | rs9648476 | A | G | 0.62 | 1.10E-08 | 32.72 |
| Tea intake | ukb-b-6066 | rs713598 | G | C | 0.4 | 5.20E-10 | 38.59 |
| Tea intake | ukb-b-6066 | rs13282783 | T | C | 0.29 | 7.90E-09 | 33.29 |
| Tea intake | ukb-b-6066 | rs56348300 | G | C | 0.18 | 6.10E-09 | 33.8 |
| Tea intake | ukb-b-6066 | rs10764990 | A | G | 0.61 | 1.90E-08 | 31.59 |
| Tea intake | ukb-b-6066 | rs10752269 | A | G | 0.51 | 1.30E-09 | 36.88 |
| Tea intake | ukb-b-6066 | rs2351187 | A | G | 0.32 | 1.60E-08 | 31.96 |
| Tea intake | ukb-b-6066 | rs17245213 | A | G | 0.21 | 2.00E-08 | 31.52 |
| Tea intake | ukb-b-6066 | rs10741694 | C | T | 0.63 | 7.90E-12 | 46.78 |
| Tea intake | ukb-b-6066 | rs1453548 | A | T | 0.66 | 3.00E-09 | 35.17 |
| Tea intake | ukb-b-6066 | rs977474 | T | C | 0.83 | 2.40E-14 | 58.18 |
| Tea intake | ukb-b-6066 | rs2783129 | G | C | 0.48 | 3.80E-08 | 30.25 |
| Tea intake | ukb-b-6066 | rs17576658 | A | G | 0.25 | 4.10E-08 | 30.12 |
| Tea intake | ukb-b-6066 | rs6829 | T | C | 0.6 | 3.70E-08 | 30.28 |
| Tea intake | ukb-b-6066 | rs2645929 | G | A | 0.81 | 3.50E-08 | 30.42 |
| Tea intake | ukb-b-6066 | rs12591786 | T | C | 0.16 | 3.70E-10 | 39.27 |
| Tea intake | ukb-b-6066 | rs2472297 | T | C | 0.26 | 2.30E-109 | 493.64 |
| Tea intake | ukb-b-6066 | rs9937354 | A | G | 0.42 | 4.90E-11 | 43.23 |
| Tea intake | ukb-b-6066 | rs9302428 | G | C | 0.64 | 2.60E-08 | 30.95 |
| Tea intake | ukb-b-6066 | rs2279844 | A | G | 0.38 | 4.00E-08 | 30.15 |
| Tea intake | ukb-b-6066 | rs4808193 | C | T | 0.34 | 1.70E-11 | 45.24 |
| Tea intake | ukb-b-6066 | rs57631352 | G | A | 0.3 | 1.70E-08 | 31.87 |
| Tea intake | ukb-b-6066 | rs2273447 | T | A | 0.2 | 3.30E-11 | 43.99 |
| Tea intake | ukb-b-6066 | rs4817505 | C | T | 0.39 | 4.20E-12 | 48.01 |
| Tea intake | ukb-b-6066 | rs132904 | C | G | 0.78 | 7.80E-11 | 42.3 |
| Tea intake | ukb-b-6066 | rs9624470 | A | G | 0.58 | 1.30E-31 | 136.84 |
| Cereal intake | ukb-b-15926 | rs10857964 | C | T | 0.21 | 1.70E-10 | 40.8 |
| Cereal intake | ukb-b-15926 | rs12354267 | C | T | 0.31 | 1.70E-09 | 36.31 |
| Cereal intake | ukb-b-15926 | rs112780312 | A | G | 0.27 | 1.80E-09 | 36.21 |
| Cereal intake | ukb-b-15926 | rs184643 | A | G | 0.57 | 1.60E-11 | 45.44 |
| Cereal intake | ukb-b-15926 | rs6545770 | T | A | 0.75 | 2.70E-11 | 44.39 |
| Cereal intake | ukb-b-15926 | rs4988235 | A | G | 0.74 | 1.50E-08 | 32.09 |
| Cereal intake | ukb-b-15926 | rs67723420 | A | T | 0.38 | 1.20E-08 | 32.49 |
| Cereal intake | ukb-b-15926 | rs7619139 | A | T | 0.59 | 9.70E-21 | 87.22 |
| Cereal intake | ukb-b-15926 | rs9846396 | T | C | 0.44 | 3.00E-11 | 44.19 |
| Cereal intake | ukb-b-15926 | rs11097340 | T | C | 0.4 | 2.10E-10 | 40.38 |
| Cereal intake | ukb-b-15926 | rs3115230 | A | C | 0.75 | 3.00E-08 | 30.71 |
| Cereal intake | ukb-b-15926 | rs11940694 | G | A | 0.6 | 5.00E-12 | 47.67 |
| Cereal intake | ukb-b-15926 | rs10057775 | C | T | 0.89 | 4.50E-12 | 47.91 |
| Cereal intake | ukb-b-15926 | rs79642906 | A | G | 0.08 | 1.90E-08 | 31.64 |
| Cereal intake | ukb-b-15926 | rs1853931 | A | G | 0.53 | 3.80E-10 | 39.22 |
| Cereal intake | ukb-b-15926 | rs6918737 | A | T | 0.23 | 7.60E-11 | 42.37 |
| Cereal intake | ukb-b-15926 | rs2817377 | A | G | 0.54 | 3.10E-08 | 30.62 |
| Cereal intake | ukb-b-15926 | rs2504706 | C | T | 0.23 | 5.30E-18 | 74.77 |
| Cereal intake | ukb-b-15926 | rs9374896 | T | C | 0.47 | 1.30E-22 | 95.69 |
| Cereal intake | ukb-b-15926 | rs4410790 | C | T | 0.63 | 3.40E-09 | 34.92 |
| Cereal intake | ukb-b-15926 | rs62442924 | T | C | 0.19 | 1.70E-08 | 31.81 |
| Cereal intake | ukb-b-15926 | rs13234131 | G | A | 0.13 | 1.60E-10 | 40.89 |
| Cereal intake | ukb-b-15926 | rs9987289 | G | A | 0.91 | 7.80E-09 | 33.34 |
| Cereal intake | ukb-b-15926 | rs4739095 | A | G | 0.77 | 9.90E-10 | 37.35 |
| Cereal intake | ukb-b-15926 | rs2927238 | G | T | 0.61 | 2.10E-08 | 31.39 |
| Cereal intake | ukb-b-15926 | rs2799849 | T | C | 0.68 | 9.80E-11 | 41.85 |
| Cereal intake | ukb-b-15926 | rs7040561 | A | T | 0.85 | 1.10E-10 | 41.68 |
| Cereal intake | ukb-b-15926 | rs491711 | C | A | 0.31 | 1.50E-09 | 36.54 |
| Cereal intake | ukb-b-15926 | rs2450126 | G | A | 0.16 | 1.30E-09 | 36.79 |
| Cereal intake | ukb-b-15926 | rs10837531 | G | C | 0.46 | 2.00E-09 | 36.02 |
| Cereal intake | ukb-b-15926 | rs11038810 | G | A | 0.64 | 2.30E-09 | 35.71 |
| Cereal intake | ukb-b-15926 | rs627185 | G | C | 0.54 | 1.50E-09 | 36.55 |
| Cereal intake | ukb-b-15926 | rs2472297 | T | C | 0.26 | 4.50E-15 | 61.47 |
| Cereal intake | ukb-b-15926 | rs1104608 | C | G | 0.43 | 2.30E-09 | 35.69 |
| Cereal intake | ukb-b-15926 | rs68136852 | A | C | 0.15 | 1.20E-08 | 32.47 |
| Cereal intake | ukb-b-15926 | rs3859193 | A | T | 0.47 | 9.50E-09 | 32.94 |
| Cereal intake | ukb-b-15926 | rs8097544 | G | A | 0.15 | 3.20E-22 | 94 |
| Cereal intake | ukb-b-15926 | rs4797242 | A | C | 0.3 | 4.50E-09 | 34.4 |
| Cereal intake | ukb-b-15926 | rs11670024 | G | A | 0.12 | 1.10E-08 | 32.71 |
| Cereal intake | ukb-b-15926 | rs6510177 | C | T | 0.81 | 1.20E-08 | 32.47 |
| Cereal intake | ukb-b-15926 | rs78854891 | C | T | 0.07 | 1.10E-09 | 37.14 |
| Cereal intake | ukb-b-15926 | rs56131196 | A | G | 0.19 | 2.70E-15 | 62.51 |
| Cereal intake | ukb-b-15926 | rs838133 | G | A | 0.55 | 3.90E-29 | 125.52 |
| Salad / raw vegetable intake | ukb-b-1996 | rs9427220 | T | A | 0.55 | 2.80E-08 | 30.83 |
| Salad / raw vegetable intake | ukb-b-1996 | rs4083969 | G | C | 0.06 | 3.80E-08 | 30.25 |
| Salad / raw vegetable intake | ukb-b-1996 | rs7619139 | A | T | 0.59 | 8.00E-18 | 73.95 |
| Salad / raw vegetable intake | ukb-b-1996 | rs13102393 | G | C | 0.5 | 2.40E-08 | 31.12 |
| Salad / raw vegetable intake | ukb-b-1996 | rs17460017 | T | A | 0.19 | 7.20E-10 | 37.97 |
| Salad / raw vegetable intake | ukb-b-1996 | rs2194027 | A | T | 0.48 | 2.00E-09 | 35.95 |
| Salad / raw vegetable intake | ukb-b-1996 | rs3129962 | A | G | 0.13 | 3.70E-10 | 39.28 |
| Salad / raw vegetable intake | ukb-b-1996 | rs12203592 | T | C | 0.22 | 1.30E-09 | 36.87 |
| Salad / raw vegetable intake | ukb-b-1996 | rs3095337 | C | G | 0.2 | 9.00E-13 | 51.06 |
| Salad / raw vegetable intake | ukb-b-1996 | rs75248709 | T | C | 0.05 | 2.20E-08 | 31.29 |
| Salad / raw vegetable intake | ukb-b-1996 | rs57221424 | G | C | 0.32 | 5.50E-09 | 34 |
| Salad / raw vegetable intake | ukb-b-1996 | rs62461186 | C | A | 0.18 | 1.00E-09 | 37.3 |
| Salad / raw vegetable intake | ukb-b-1996 | rs790561 | G | A | 0.7 | 1.40E-15 | 63.78 |
| Salad / raw vegetable intake | ukb-b-1996 | rs7821179 | C | G | 0.85 | 4.40E-08 | 29.96 |
| Salad / raw vegetable intake | ukb-b-1996 | rs10819082 | A | G | 0.67 | 1.40E-09 | 36.67 |
| Salad / raw vegetable intake | ukb-b-1996 | rs6482190 | G | A | 0.72 | 1.40E-12 | 50.24 |
| Salad / raw vegetable intake | ukb-b-1996 | rs1890012 | G | T | 0.19 | 8.10E-09 | 33.25 |
| Salad / raw vegetable intake | ukb-b-1996 | rs12908495 | A | C | 0.24 | 2.00E-08 | 31.51 |
| Salad / raw vegetable intake | ukb-b-1996 | rs1052352 | T | C | 0.52 | 1.00E-08 | 32.8 |
| Salad / raw vegetable intake | ukb-b-1996 | rs34186148 | C | G | 0.37 | 4.80E-08 | 29.8 |
| Salad / raw vegetable intake | ukb-b-1996 | rs4291983 | A | C | 0.52 | 3.70E-09 | 34.76 |
| Salad / raw vegetable intake | ukb-b-1996 | rs8130508 | A | G | 0.29 | 3.00E-08 | 30.68 |
| Coffee intake | ukb-b-5237 | rs516636 | A | C | 0.21 | 4.00E-09 | 34.63 |
| Coffee intake | ukb-b-5237 | rs4615895 | A | G | 0.74 | 4.20E-11 | 43.52 |
| Coffee intake | ukb-b-5237 | rs13387939 | A | C | 0.83 | 9.80E-15 | 59.93 |
| Coffee intake | ukb-b-5237 | rs780093 | C | T | 0.62 | 1.00E-15 | 64.37 |
| Coffee intake | ukb-b-5237 | rs12989746 | T | G | 0.25 | 2.80E-08 | 30.82 |
| Coffee intake | ukb-b-5237 | rs1527961 | C | T | 0.13 | 1.70E-08 | 31.81 |
| Coffee intake | ukb-b-5237 | rs2597805 | T | C | 0.68 | 2.00E-08 | 31.49 |
| Coffee intake | ukb-b-5237 | rs2189234 | G | T | 0.62 | 1.80E-09 | 36.17 |
| Coffee intake | ukb-b-5237 | rs13163336 | A | C | 0.16 | 1.30E-11 | 45.74 |
| Coffee intake | ukb-b-5237 | rs12514566 | A | G | 0.34 | 2.40E-11 | 44.65 |
| Coffee intake | ukb-b-5237 | rs2465037 | A | C | 0.34 | 4.80E-10 | 38.77 |
| Coffee intake | ukb-b-5237 | rs1338549 | G | T | 0.53 | 5.60E-09 | 33.96 |
| Coffee intake | ukb-b-5237 | rs9398171 | T | C | 0.71 | 1.10E-09 | 37.21 |
| Coffee intake | ukb-b-5237 | rs73075167 | T | A | 0.13 | 5.00E-11 | 43.19 |
| Coffee intake | ukb-b-5237 | rs7811609 | T | C | 0.37 | 4.00E-08 | 30.14 |
| Coffee intake | ukb-b-5237 | rs1057868 | T | C | 0.28 | 5.40E-29 | 124.9 |
| Coffee intake | ukb-b-5237 | rs4410790 | C | T | 0.63 | 1.20E-120 | 545.51 |
| Coffee intake | ukb-b-5237 | rs34060476 | G | A | 0.13 | 7.50E-15 | 60.45 |
| Coffee intake | ukb-b-5237 | rs6469262 | C | T | 0.56 | 1.90E-08 | 31.58 |
| Coffee intake | ukb-b-5237 | rs78267637 | G | C | 0.04 | 3.90E-09 | 34.7 |
| Coffee intake | ukb-b-5237 | rs442355 | C | G | 0.25 | 1.90E-09 | 36.1 |
| Coffee intake | ukb-b-5237 | rs10119174 | C | G | 0.57 | 1.00E-08 | 32.77 |
| Coffee intake | ukb-b-5237 | rs117810762 | A | G | 0.02 | 6.20E-09 | 33.78 |
| Coffee intake | ukb-b-5237 | rs61928609 | C | A | 0.84 | 1.30E-11 | 45.85 |
| Coffee intake | ukb-b-5237 | rs2472297 | T | C | 0.26 | 1.10E-142 | 646.73 |
| Coffee intake | ukb-b-5237 | rs117968677 | A | G | 0.02 | 1.90E-08 | 31.65 |
| Coffee intake | ukb-b-5237 | rs8056750 | T | C | 0.36 | 1.30E-09 | 36.78 |
| Coffee intake | ukb-b-5237 | rs1421085 | C | T | 0.4 | 1.70E-29 | 127.16 |
| Coffee intake | ukb-b-5237 | rs62064918 | T | C | 0.24 | 4.10E-08 | 30.1 |
| Coffee intake | ukb-b-5237 | rs57918684 | A | G | 0.15 | 8.60E-09 | 33.14 |
| Coffee intake | ukb-b-5237 | rs7224815 | T | A | 0.41 | 3.70E-11 | 43.77 |
| Coffee intake | ukb-b-5237 | rs630194 | C | T | 0.34 | 2.30E-11 | 44.68 |
| Coffee intake | ukb-b-5237 | rs1942965 | C | T | 0.5 | 3.80E-08 | 30.24 |
| Coffee intake | ukb-b-5237 | rs476828 | C | T | 0.24 | 5.60E-20 | 83.75 |
| Coffee intake | ukb-b-5237 | rs56113850 | C | T | 0.58 | 8.90E-15 | 60.13 |
| Coffee intake | ukb-b-5237 | rs75347775 | A | G | 0.24 | 2.70E-08 | 30.93 |
| Coffee intake | ukb-b-5237 | rs6063085 | C | A | 0.37 | 4.50E-10 | 38.9 |
| Coffee intake | ukb-b-5237 | rs6062682 | T | C | 0.46 | 2.50E-10 | 40.02 |
| Coffee intake | ukb-b-5237 | rs13054099 | C | T | 0.26 | 4.30E-09 | 34.46 |
| Coffee intake | ukb-b-5237 | rs17842490 | G | A | 0.01 | 3.30E-11 | 44.01 |
| Dried fruit intake | ukb-b-16576 | rs261809 | G | A | 0.54 | 9.80E-09 | 32.89 |
| Dried fruit intake | ukb-b-16576 | rs11586016 | C | G | 0.37 | 1.10E-08 | 32.59 |
| Dried fruit intake | ukb-b-16576 | rs12137234 | T | C | 0.3 | 2.80E-08 | 30.85 |
| Dried fruit intake | ukb-b-16576 | rs72720396 | G | A | 0.23 | 8.70E-09 | 33.12 |
| Dried fruit intake | ukb-b-16576 | rs11811826 | A | T | 0.22 | 4.40E-11 | 43.43 |
| Dried fruit intake | ukb-b-16576 | rs3101339 | C | A | 0.6 | 6.20E-17 | 69.91 |
| Dried fruit intake | ukb-b-16576 | rs75641275 | C | A | 0.14 | 2.90E-09 | 35.25 |
| Dried fruit intake | ukb-b-16576 | rs7582086 | T | G | 0.47 | 8.80E-09 | 33.09 |
| Dried fruit intake | ukb-b-16576 | rs7599488 | T | C | 0.43 | 6.70E-10 | 38.1 |
| Dried fruit intake | ukb-b-16576 | rs4149513 | A | G | 0.49 | 2.20E-12 | 49.25 |
| Dried fruit intake | ukb-b-16576 | rs17184707 | T | C | 0.21 | 2.10E-08 | 31.43 |
| Dried fruit intake | ukb-b-16576 | rs4269101 | G | T | 0.72 | 1.10E-13 | 55.17 |
| Dried fruit intake | ukb-b-16576 | rs11720884 | G | A | 0.25 | 7.60E-09 | 33.36 |
| Dried fruit intake | ukb-b-16576 | rs57499472 | C | T | 0.4 | 8.10E-09 | 33.26 |
| Dried fruit intake | ukb-b-16576 | rs10026792 | A | G | 0.29 | 3.90E-09 | 34.66 |
| Dried fruit intake | ukb-b-16576 | rs1648404 | T | C | 0.48 | 1.80E-08 | 31.65 |
| Dried fruit intake | ukb-b-16576 | rs746868 | G | C | 0.61 | 5.20E-14 | 56.66 |
| Dried fruit intake | ukb-b-16576 | rs9385269 | T | C | 0.52 | 7.20E-13 | 51.48 |
| Dried fruit intake | ukb-b-16576 | rs2328887 | C | T | 0.9 | 8.80E-12 | 46.59 |
| Dried fruit intake | ukb-b-16576 | rs2533273 | A | C | 0.48 | 3.90E-09 | 34.68 |
| Dried fruit intake | ukb-b-16576 | rs7808471 | C | T | 0.32 | 1.10E-10 | 41.72 |
| Dried fruit intake | ukb-b-16576 | rs11772627 | C | G | 0.18 | 3.00E-17 | 71.32 |
| Dried fruit intake | ukb-b-16576 | rs7829800 | G | A | 0.67 | 5.10E-09 | 34.17 |
| Dried fruit intake | ukb-b-16576 | rs10740991 | C | G | 0.72 | 2.00E-19 | 81.23 |
| Dried fruit intake | ukb-b-16576 | rs7916868 | T | A | 0.5 | 9.10E-09 | 33.03 |
| Dried fruit intake | ukb-b-16576 | rs893856 | A | G | 0.15 | 1.30E-08 | 32.35 |
| Dried fruit intake | ukb-b-16576 | rs10896126 | G | A | 0.3 | 1.60E-16 | 68.07 |
| Dried fruit intake | ukb-b-16576 | rs11037497 | C | G | 0.45 | 5.70E-10 | 38.41 |
| Dried fruit intake | ukb-b-16576 | rs1622515 | G | A | 0.48 | 2.90E-09 | 35.23 |
| Dried fruit intake | ukb-b-16576 | rs3764002 | T | C | 0.26 | 5.10E-12 | 47.65 |
| Dried fruit intake | ukb-b-16576 | rs4140799 | A | G | 0.53 | 1.80E-08 | 31.74 |
| Dried fruit intake | ukb-b-16576 | rs34162196 | T | C | 0.1 | 7.10E-16 | 65.1 |
| Dried fruit intake | ukb-b-16576 | rs10129747 | G | A | 0.53 | 2.60E-08 | 30.99 |
| Dried fruit intake | ukb-b-16576 | rs1797235 | C | G | 0.37 | 8.90E-09 | 33.08 |
| Dried fruit intake | ukb-b-16576 | rs11632215 | C | A | 0.12 | 4.40E-08 | 29.96 |
| Dried fruit intake | ukb-b-16576 | rs862227 | G | A | 0.46 | 4.30E-08 | 30.03 |
| Dried fruit intake | ukb-b-16576 | rs1582322 | G | A | 0.6 | 6.80E-09 | 33.59 |
| Dried fruit intake | ukb-b-16576 | rs62084586 | C | T | 0.17 | 3.20E-09 | 35.07 |
| Dried fruit intake | ukb-b-16576 | rs8081370 | T | C | 0.91 | 1.40E-08 | 32.18 |
| Dried fruit intake | ukb-b-16576 | rs4800488 | A | C | 0.49 | 7.70E-13 | 51.37 |
| Dried fruit intake | ukb-b-16576 | rs17175518 | A | C | 0.23 | 5.90E-09 | 33.88 |
| Dried fruit intake | ukb-b-16576 | rs11152349 | A | G | 0.3 | 4.90E-08 | 29.74 |
| Dried fruit intake | ukb-b-16576 | rs429358 | C | T | 0.15 | 6.70E-18 | 74.31 |

Abbreviations: MR: Mendelian randomization; SNP: single nucleotide polymorphisms.

Table S2. The International Classification of Diseases (ICD) diagnosis codes used to define cases with hypertension and controls in the FinnGen Study

| Diagnostic classification codes |  | Individuals with hypertension |
| --- | --- | --- |
|  | ICD-8 | 401\|402\|403\|404 |
|  | ICD-9 | 4039A\| 4019X |
|  | ICD-10 | \|10 |


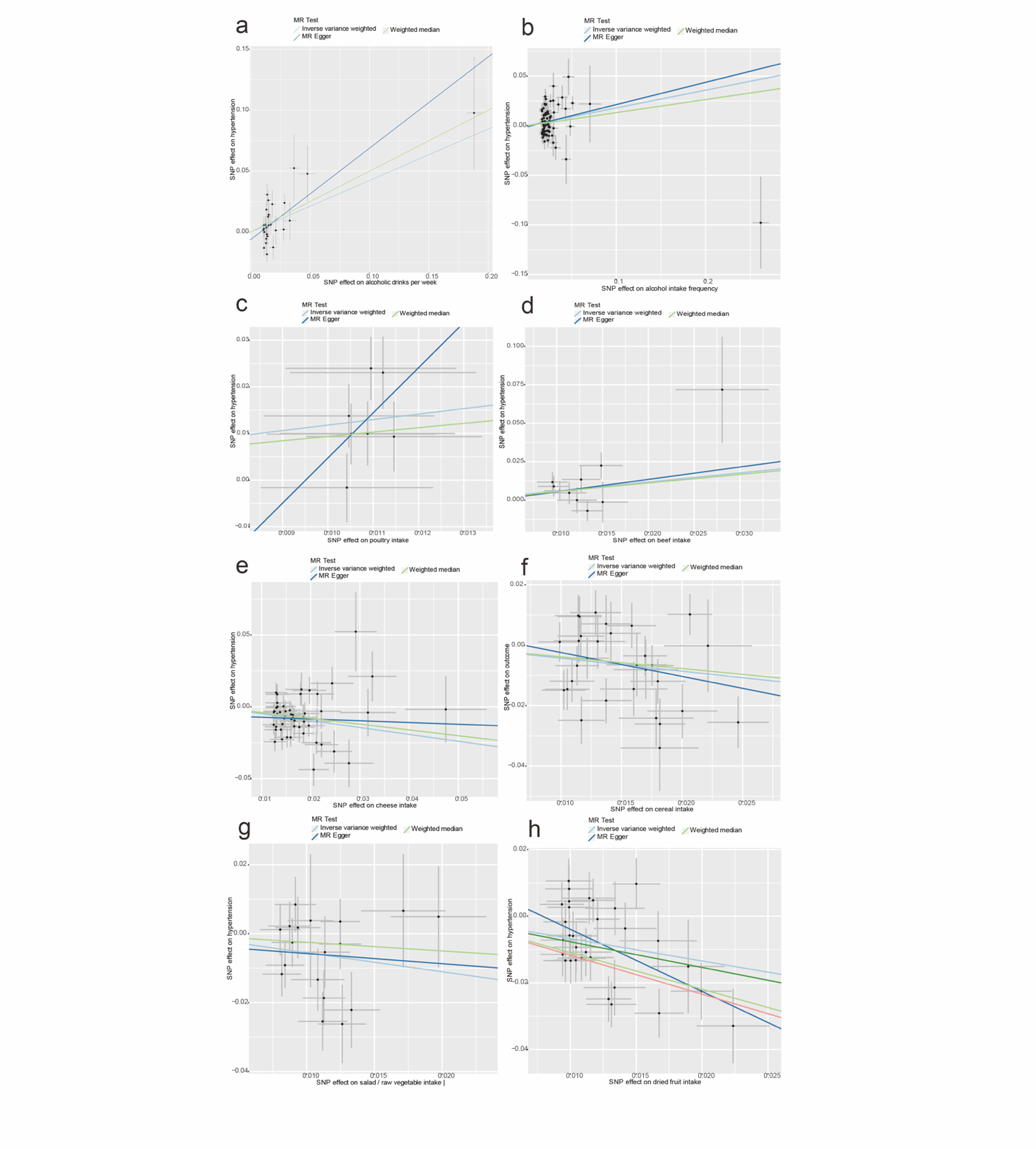


Figure S1. Scatter plot of the significant association of dietary factors with hypertension. (a) alcohol drinks per week; (b) alcohol intake frequency; (c) poultry intake; (d) beef intake; (e) cheese intake; (f) cereal intake; (g) salad/raw vegetable intake; (h) dried fruit intake.


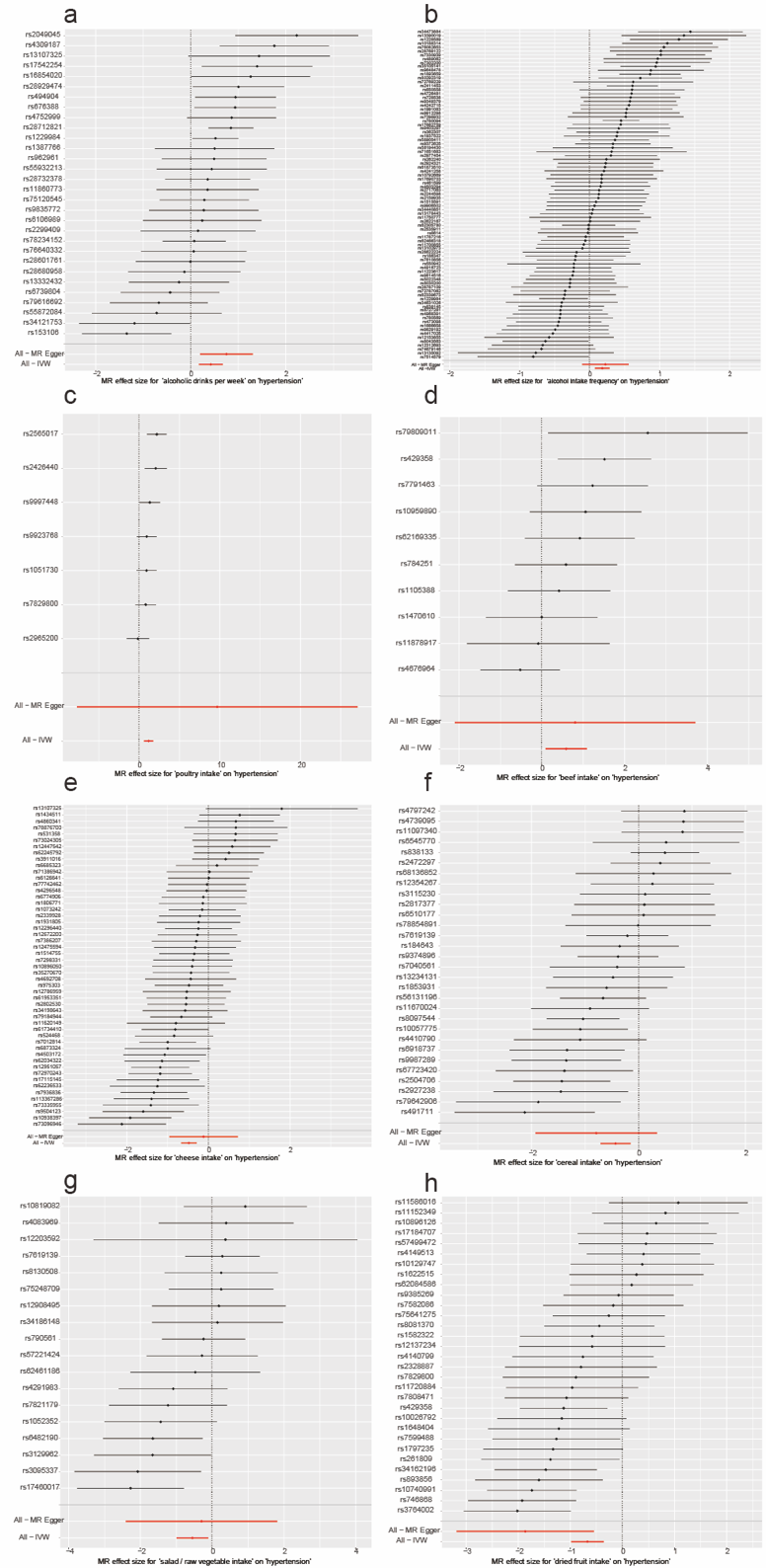


Figure S2. Forest plot of the significant association of dietary factors with hypertension. (a) alcohol drinks per week; (b) alcohol intake frequency; (c) poultry intake; (d) beef intake; (e) cheese intake; (f) cereal intake; (g) salad/raw vegetable intake; (h) dried fruit intake.


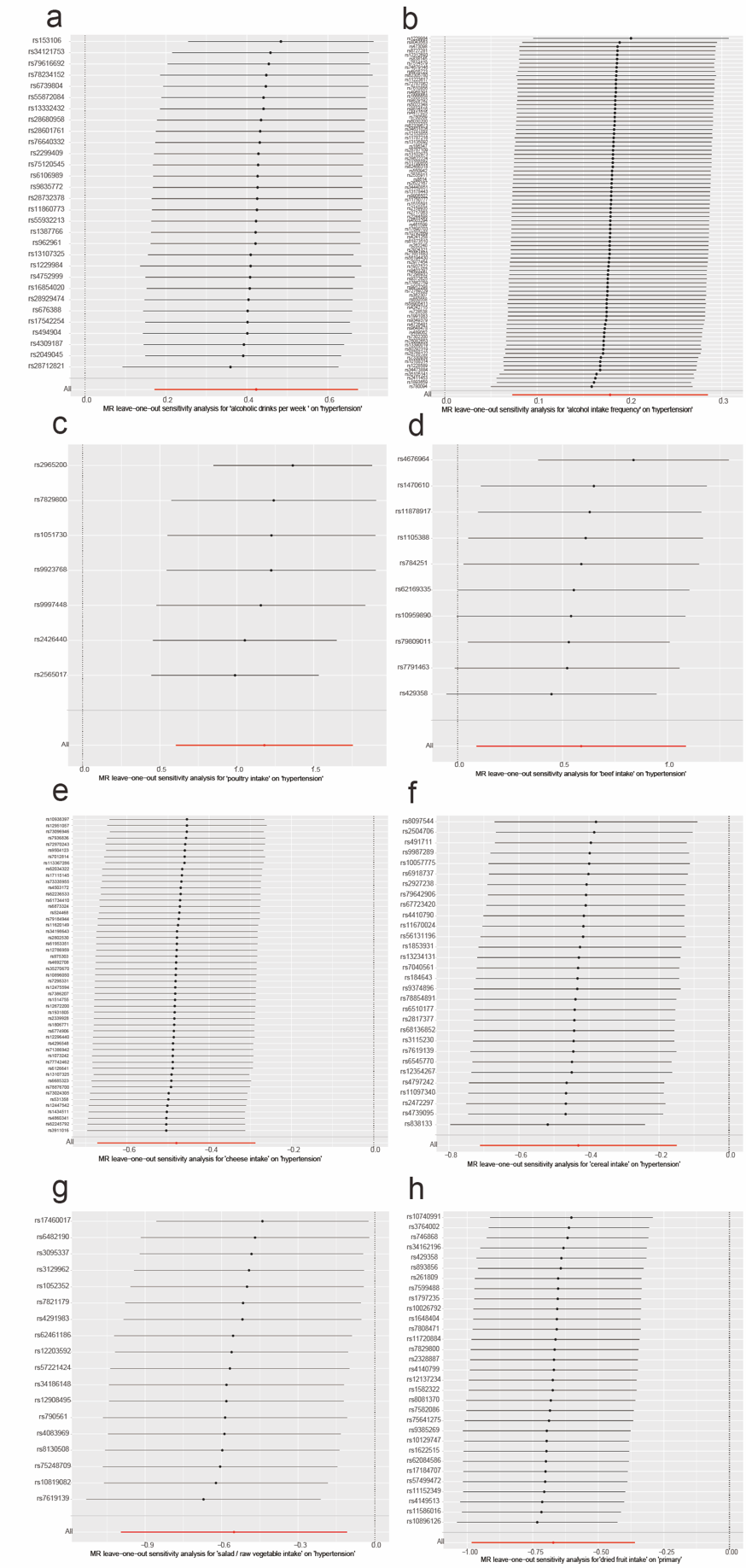


Figure S3. Leave-one-out sensitivity analysis of the significant association of dietary factors with hypertension. (a) alcohol drinks per week; (b) alcohol intake frequency; (c) poultry intake; (d) beef intake; (e) cheese intake; (f) cereal intake; (g) salad/raw vegetable intake; (h) dried fruit intake.


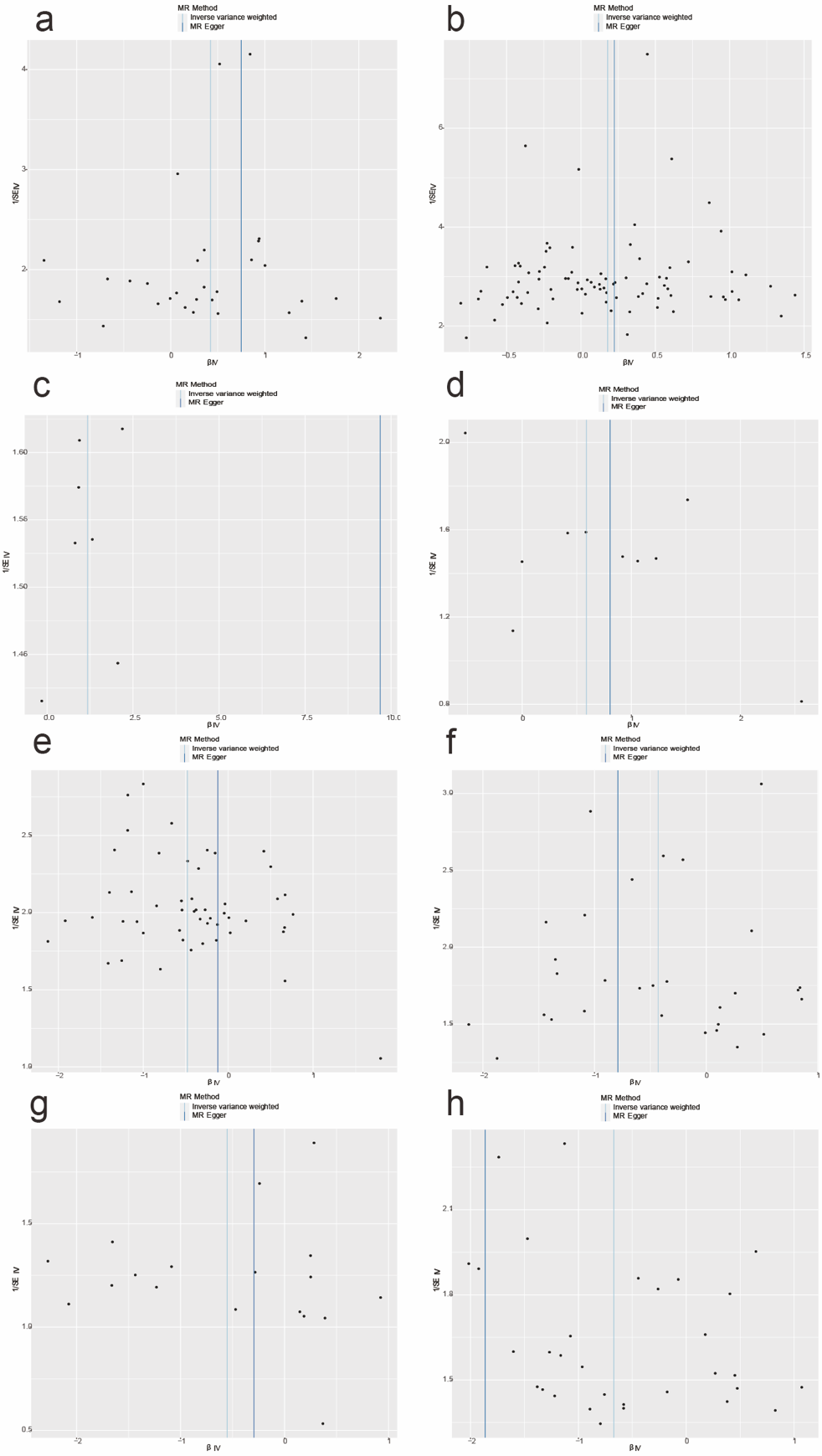


Figure S4. Funnel plot of the significant association of dietary factors with hypertension. (a) alcohol drinks per week; (b) alcohol intake frequency; (c) poultry intake; (d) beef intake; (e) cheese intake; (f) cereal intake; (g) salad/raw vegetable intake; (h) dried fruit intake.
